# What is the state of the art on traditional medicine interventions for zoonotic diseases in the Indian subcontinent? A scoping review of the peer-reviewed evidence base

**DOI:** 10.1186/s12906-024-04553-8

**Published:** 2024-06-29

**Authors:** Festus A. Asaaga, Emmanuel S. Tomude, Mujeeb Rahman, Irfan Shakeer, Nitya S. Ghotge, Sarah J. Burthe, Stefanie M. Schäfer, Abi T. Vanak, Bethan V. Purse, Subhash L. Hoti

**Affiliations:** 1 UK Centre for Ecology & Hydrology, Wallingford, United Kingdom OX10 8BB; 2 Ashoka Trust for Research in Ecology and the Environment, Bengaluru, India; 3 Anthra, Lantana Gardens NDA Road, Bavdhan, Pune, 411021 Maharashtra India; 4 UK Centre for Ecology & Hydrology, Edinburgh, United Kingdom EH26 0QB; 5 ICMR-Vector Control Research Centre, Puducherry, India; 6 School of Life Sciences, University of KwaZulu-Natal, Durban, South Africa

**Keywords:** Traditional medicine, Traditional medicine interventions, Zoonotic diseases, Scoping review, Indian subcontinent

## Abstract

**Background:**

Traditional medicine (TM) interventions are plausible therapeutic alternatives to conventional medical interventions against emerging and endemic zoonotic diseases, particularly in low-and middle-income countries that may lack resources and infrastructure. Despite the growing popularity in the usage of TM interventions, their clinical safety and effectiveness are still contested within conventional healthcare in many countries.

**Methods:**

We conducted a scoping review of the peer-reviewed literature that synthesises and maps the evidence on TM interventions for the treatment and prevention of zoonoses on the Indian subcontinent. The region, a global hotspot of biodiversity and emerging infections, is characterised by high prevalence of TM use. Based on the scientific literature (mostly case study research, *n*=l06 studies), our review (1) maps the scope of the literature, (2) synthesises the evidence on the application of TM interventions for zoonoses, and (3) critically reflects on the state of TM and identifies areas for future research focus.

**Results:**

The evidence synthesis confirmed widespread usage of TM interventions for zoonoses on the subcontinent, with the majority of research reported from India (*n*=99 studies, 93.4%), followed by Pakistan (*n*=3 studies, 2.8%), Bangladesh (*n*=2 studies, 1.9%), and Sri Lanka (*n*=1, 0.9%). Most of the reviewed studies reported on ethno-medicinal uses of plant species, primarily for treating dengue (*n*=20 studies), tuberculosis (*n*=18 studies), *Escherichia coli* infection (*n*=16 studies), lymphatic filariasis and cholera (*n*=9 apiece). However, the evidence on the safety and effectiveness of these reported TM interventions is limited, indicating that these data are rarely collected and/or shared within the peer-reviewed literature.

**Conclusion:**

This review thus highlights that, whilst TMs are already being used and could offer more widely accessible interventions against emerging and endemic zoonoses and ectoparasites, there is an urgent need for rigorous clinical testing and validation of the safety and effectiveness of these interventions.

**Supplementary Information:**

The online version contains supplementary material available at 10.1186/s12906-024-04553-8.

## Background

Emerging and endemic zoonotic diseases (caused by pathogens passed between animals and people) are increasing globally and posing massive threats to health and wellbeing. Hence there is huge drive to focus health policy and practice to effectively address these challenges, particularly in low-and middle-income countries (LMICs). Despite the fact that emerging and endemic zoonotic diseases often have huge disproportionate impacts on vulnerable and poor segment of the population in LMICs [1], such countries often lack the requisite resources and healthcare infrastructure to deal with them [2, 3]. This has also brought into the spotlight and generated debate about the potential role of traditional medicine (TM hereafter) interventions as alternative treatments to conventional medicine in LMICs. Traditional medicine, in this context is “the sum total of the knowledge, skills and practices based on the theories, beliefs and experiences indigenous to different cultures, whether explicable or nor, used in the maintenance of health as well as the prevention, diagnosis, improvement, or treatment of [zoonotic-related] illnesses” following the definition of the World Health Organisation [4]. TM therapies and related practices often involve the use of plants, animals (zoo-therapeutics) and other derivative materials as principal source ingredients in their preparation [5].

It is common knowledge that rural and indigenous populations (with limited access to formal healthcare infrastructure) in LMICs have relied particularly on TM therapies since time immemorial [5, 6]. It has been estimated that approximately 80% of the populations in LMICs depend on TM for their primary healthcare [7, 8]. However, the effectiveness, clinical safety and utility of related TM practices are still contested within the spheres of contemporary healthcare in many jurisdictions, including the Indian subcontinent [9–11]. Misconceptions and counter claims about effectiveness and safety of TM interventions have often hindered their mainstreaming or formal acceptance into conventional medical practice [12]. This is despite the fact some “traditional medicines” have led to the development of conventional medicines (e.g. Ayurvedic-based food supplements) that are now used globally [13].

A considerable body of literature highlights the importance of recognising the use-value and/or beneficence of TM interventions [6, 12, 14, 15], especially in the treatment of emerging zoonotic diseases, including Covid-19 [16], dengue and chikungunya [17–19]. Elsewhere, in sub-Saharan African countries such as Ghana, Mali, Nigeria, and Zambia, the first line of treatment for 60% of the children with malarial related high fevers, is the use of home-based TMs [20]. Following the Alma Atta declaration in 1978, the WHO recommended the inclusion of TM and related medicinal drugs in primary healthcare [21]. The growing recognition of the value to preserve ethnobotanical and ethno-zoological knowledge systems and related TM practices along with the renewed interest of global pharmaceutical companies in natural product development [2, 8, 22], gives further impetus for systematic investigation of the role of TM for controlling infectious diseases in the contemporary contexts. Recent studies have begun to characterise the evidence base on TM interventions for the treatment and control of specific zoonotic diseases such as lymphatic filariasis [23], rabies [3] and tuberculosis [14] across different geographical contexts.

Despite the valuable contributions of these and other previous studies, there are some notable gaps in our understanding of the role of traditional medicine (TM) interventions for treating zoonotic diseases. First, much of the existing research on TM remedies for treating zoonotic diseases has narrowly focussed on localised case studies and populations, limiting generalizable insights at a broader landscape or regional scale. Therefore, there is a need for secondary studies that can aggregate, analyse and interpret the results of empirical research. Such aggregation of empirical studies is useful for prioritisation among and building evidence of effectiveness of potential treatments in different contexts. Second, we know little about the (perceived or actual) effectiveness and potential adverse effects of TM interventions for zoonotic diseases and ectoparasites (i.e. vectors of pathogens involved in the transmission of various zoonotic diseases) in different socio-spatial contexts [2, 23]. This is especially true in the case of emerging zoonotic diseases which are on the rise [24, 25] and are characterised by marked variations in their socio-economic burden, patterns of vulnerability, health-seeking behaviours and adaptation pathways within and across socio-spatial contexts [1, 26, 27]. This study aims to contribute to the evidence base on the role of TM interventions for (re-)emerging zoonotic diseases by developing a comprehensive database of TM interventions for the treatment and prevention of zoonotic diseases reported from the Indian subcontinent. Third, as the research area of the TM systems and emerging infectious diseases evolves and the number of studies increases, there is a need to systematically identify, analyse and classify the state-of-the-art of this research area. Despite the sub-region’s long history of TM practice through Ayurveda, Unani, and Siddha systems of medicine [13, 23, 28], to the best of our knowledge, there is no systematic synthesis that critically evaluates available evidence on ethnobotanical and/or ethno-zoological knowledge and practices (e.g. ayurveda) utilised by local and tribal populations for the purposes of treating and controlling emerging and endemic zoonotic diseases. The Indian subcontinent is one of the global hotspots for (re-)emerging zoonotic diseases with high burdens [29]. The region hosts significant diversity of fauna and flora that contribute to its ethno-pharmacopeia and therapeutic patrimony, and the widespread usage of TMs for the treatment and prevention of various communicable diseases [30].

In this paper, we aim to map and synthesise existing evidence on TM interventions for zoonotic diseases in the Indian subcontinent, with a view to identify current level of evidence and significance of these interventions, the key gaps in our knowledge as well as avenues for future research. Specifically, we (1) map and synthesise the evidence on TM usage and effectiveness (i.e. reduction in symptom severity and/or mortality in infected individuals) for treatment and prevention of zoonotic diseases in the Indian subcontinent, and (2) identify key avenues for future research and practice.

## Materials and methods

This study systematically maps the evidence base on the application of TM interventions for treatment of zoonotic diseases and related knowledge gaps in the scientific literature, following the method proposed by Arksey and O’Malley [31], as well as the Preferred Reporting Items for Systematic Reviews and Meta-Analyses extension for scoping reviews (PRISMA-ScR) [32].

### Identifying the research questions

We addressed the overarching research question, “what has been studied about TM interventions for treatment and prevention of zoonotic diseases?”. The corpus of studies for the review included all primary research on traditional interventions to the subject-matter in our defined geographical area, the Indian subcontinent. Specifically, we addressed the sub-questions: (1) What kind of research has been conducted? (2) Which TM remedies are mainly used for which zoonotic diseases and what are the modes of application? (3) What is the level of evidence for the effectiveness of TM interventions for treatment of zoonotic diseases?

### Search strategy

Given their wide scope of scientific publications and multidisciplinary contents [26], we searched four online bibliographic databases (Web of Science, PubMed, Scopus and Google Scholar) for relevant peer-reviewed articles. In addition, we searched Indian scientific databases for peer-reviewed articles by inspecting the first two pages (approximately 100 results per page) of results and then subsequently screening the following two pages or until no more relevant results were found. Our search strategy comprised of two key steps – preliminary search and main search. The preliminary search was to facilitate the construction and review of different trail search strings to inform the main search. Following the preliminary search, we performed a database keyword search during the main search to retrieve relevant studies in four electronic databases listed in Table S1.

The resultant search results from the preliminary and main searches were amalgamated in an Excel spreadsheet and excluded duplicate studies for the title and abstract level screening of relevant papers. In addition, we searched Google Scholar for grey literature and non-indexed studies by inspecting the first 300 studies of results to identify relevant publications. There was no timeline restriction for the database searches. The reference lists of randomly selected articles were manually searched with a “snowball” technique utilized to identify any further literature that may have been missed in the first search round until saturation of the search has been reached. We executed the search between August 2022 and December 2022. The key terms applied in the search are summarised in the supplementary excel spreadsheet in Table S2.

### Review criteria and study selection

**Fig. 1:**
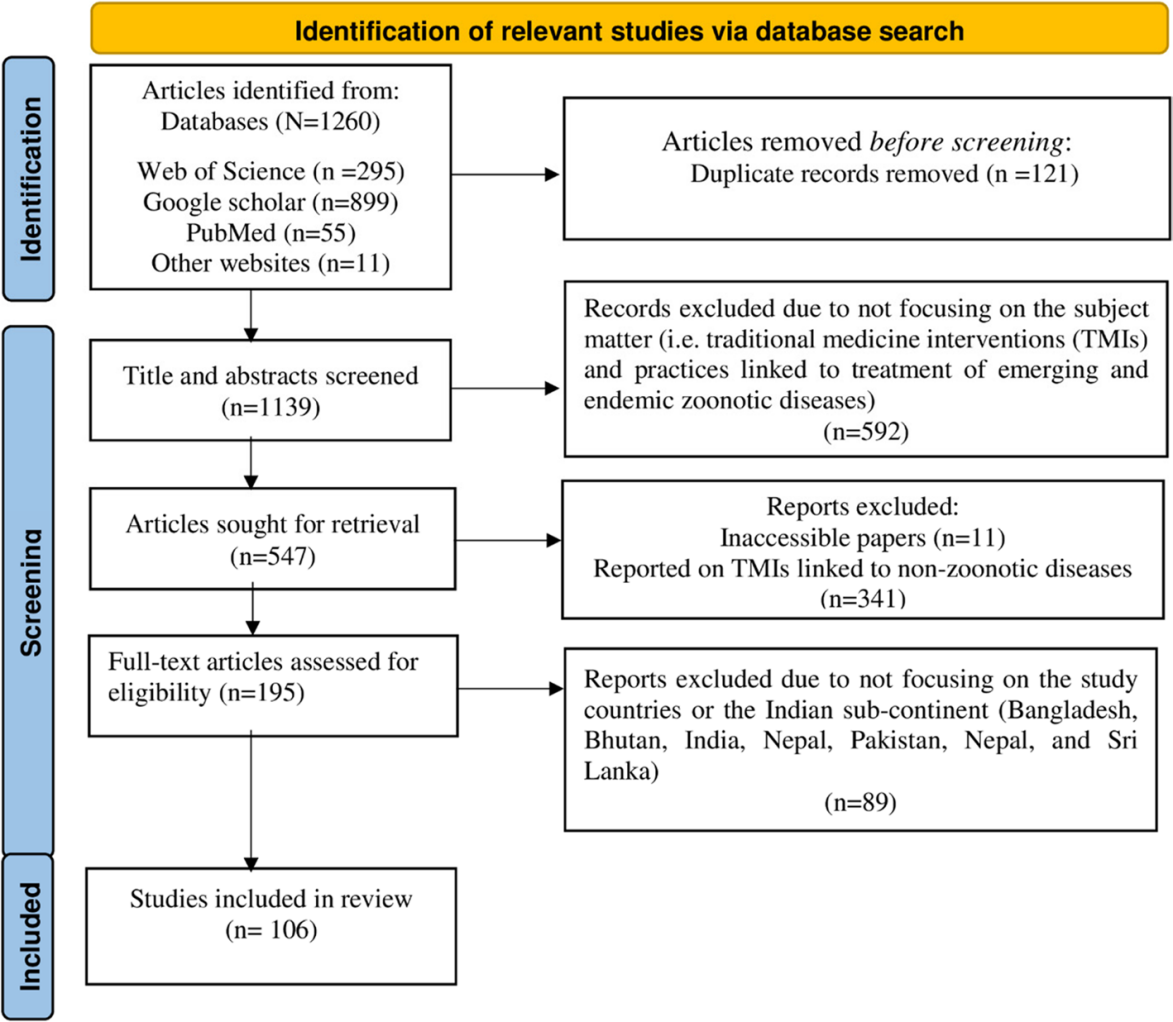
Flowchart of the selection process of relevant articles. Adapted from the Preferred Reporting Items for Systematic Review and Meta-Analysis (PRISMA) protocol by Page et al. [32]

Following the systematic search, we considered all 1260 studies for the subsequent study selection comprising of two screening phases: (1) selection of relevant articles based on their title and abstract, and (2) selection of relevant studies based on their full text. The study selection was based on a defined inclusion and exclusion criteria and was conducted by two reviewers (MR & IS) in parallel. A third reviewer (EST) provided an oversight for the screening process and selection results were harmonised through team discussions. To delimit the scope of our review to subject-relevant papers and minimise the likelihood of bias, we developed a set of inclusion/exclusion criteria to screen abstracts of the search results. These were discussed, piloted and validated by four reviewers (FAA, MR, IS & EST) prior to the screening exercise. The main criteria for inclusion were that studies focus on at least one TM intervention and the focal disease must be zoonotic (transmittable from animals to humans). In addition, we concentrated on subject-relevant studies written in the English language and focussed on India or the Indian subcontinent. In total, 106 studies were tagged as relevant to the focus of our study after team discussion with all authors and resolving any conflicts. 1,022 papers were found to be out of the scope of our review. These included papers that did not discuss TM interventions for the treatment and/or control of zoonoses in the Indian subcontinent (Fig. 1). Table 1 summarises the inclusion/exclusion criteria applied to screening eligible studies.

**Table 1:** Inclusion and exclusion criteria

**Code**	**Criteria**	**Assessment criteria**
I1	Inclusion	Primarily focussed on the subject matter – indigenous or traditional medicine knowledge and practices related to zoonotic diseases
I2	Inclusion	Reporting on ethno-medicinal and/or zoo-therapeutic knowledge, traditional medicine interventions or practitioners within the context of treatment of zoonotic diseases
I3	Inclusion	Peer-reviewed, i.e. published in scientific journals, conference or workshop proceedings.
I4	Inclusion	Geographical focus on India or the Indian subcontinent
E1	Exclusion	Studies that do not report on indigenous or traditional medicine knowledge and healing practices linked to the treatment and/or control of zoonotic diseases.
E2	Exclusion	Studies reporting on ethnobotanical, plant physiology, and reviews of specific indigenous plants, as they are most often not based on the context of traditional medicine but the function and action of the plant itself.
E3	Exclusion	Non-English studies
E4	Exclusion	Not available as a full text.

### Data extraction, coding and analysis

To facilitate the ease of data extraction and management, we developed a coding framework in Bristol Online Survey (BOS) for scoring relevant studies. The full text of all relevant publications was obtained and organised in an online reference manager (Sciwheel) in readiness for data extraction. To minimise bias in the data extraction results, three reviewers (MR, IS & EST) performed the data extraction independently. Prior to the actual data extraction exercise, the authors discussed the definitions of the data items to be extracted followed by a pilot scoring of five studies selected at random to ensure a shared understanding. The BOS form had the following headings: (1) source identifiers (lead author, publication year), (2) source characteristics (i.e. disease focus, country, primary objective, study design, study population, key findings in relation to the subject matter. We also extracted the metadata on each document (i.e. author, title, journal year and funding details) and the study methods and geographical location(s). The extracted data screen the documents via a thematic and content analysis approach [33] to specifically identify relevant data items such as specific TM interventions, application, knowledge holders, related disease conditions etc. as captured in the coding framework. We performed a descriptive statistic (frequency and percentage) of reported TM intervention characteristics, including target social groups, zoonotic disease conditions.

## Results and synthesis

We present the main findings and analysis of the scientific literature. We first synthesise the literature landscape on the application of TMIs for treatment and control of zoonotic diseases, the geographical coverage and representation.

### Current state of the literature landscape

**Fig. 2:**
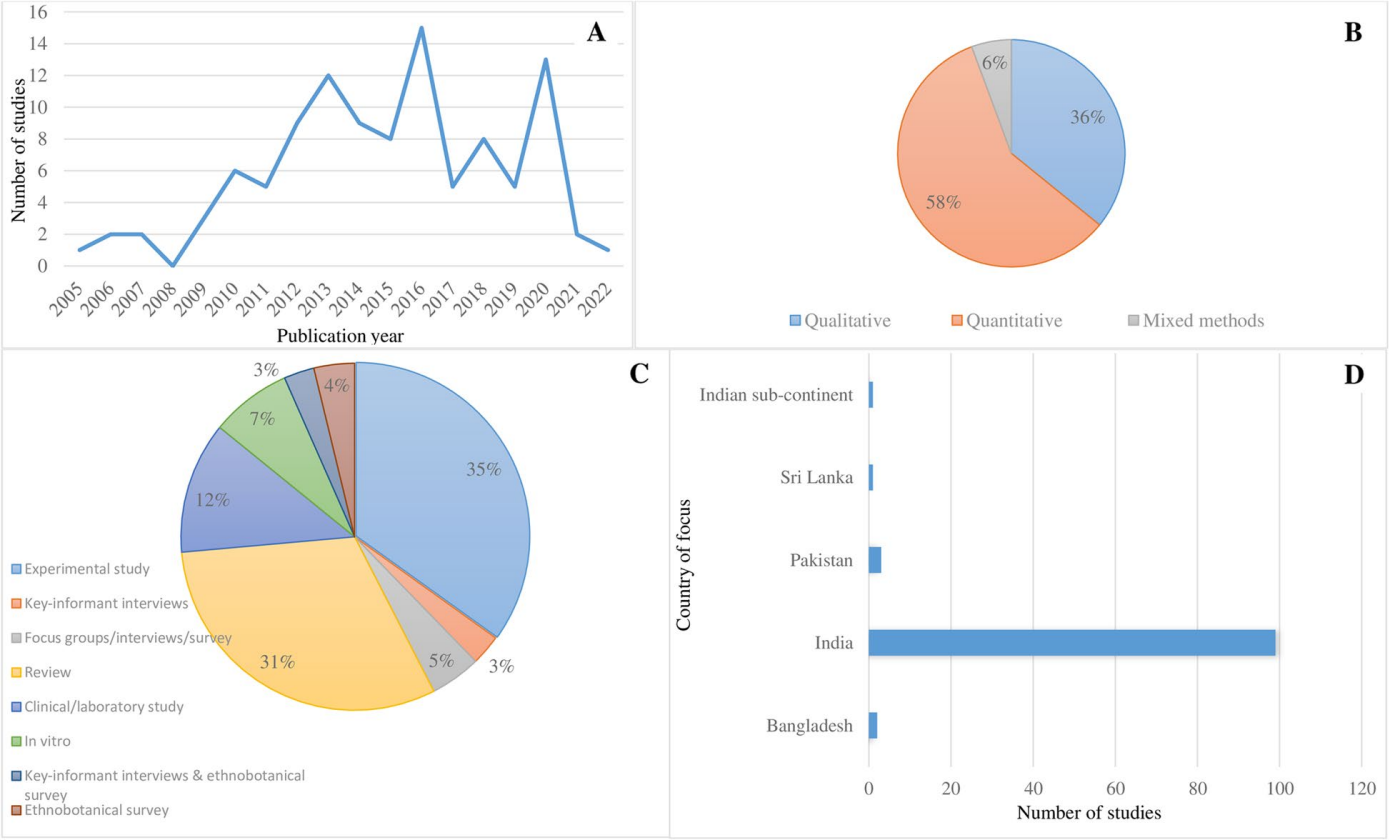
Descriptive summary of included studies. **A** For relevant abstracts, trends in publication over time indicate a continued increase in the volume of literature on traditional medicine (TM) interventions for zoonotic diseases in the Indian subcontinent. Literature published between 1^st^ January 2005, and December 2022 were included. Pie charts show the number of publications by study design (**B**) and specific data collation techniques used (**C**). (**D**) Study countries – each represented country mentioned in at least one study

We retrieved a total of 1260 relevant citations from four databases, after removal of duplicates, and 1139 citations were selected for the abstract and full-text screening. After excluding 89 full texts, the final sample consisted of 106 subject-relevant studies which met all the inclusion criteria (Fig. 1). The included studies were published from 2005 to 2022, with a significant increase in publication in 2016 and 2020, the later spike possibly due to the influences of the Covid-19 pandemic. While the study designs and methods were not always clearly described, 62 studies (58.5%) were quantitative (mostly ethnobotanical surveys), 38 (35.8%) qualitative (e.g. key-informant interviews, textual analysis), and 6 (5.7%) mixed methods. On the geographical distribution of subject-relevant studies, the majority reported research from India (*n*=99 studies, 93.4%), followed by Pakistan (*n*=3 studies, 2.8%), Bangladesh (*n*=2 studies, 1.9%), and Sri Lanka (*n*=1, 0.9%) respectively (Fig. 2). There was one multi-country study. The included studies were published in a diverse corpus of refereed journals, with no clearly discernible trends in journal preferences. Figure 1 illustrates the article selection process and included studies in the review.

A further bibliometric analysis of the included studies shows that 10 studies (representing 9.4%) involved international collaborations, implying that one or more of the co-authors reported institutional affiliations outside of the Indian subcontinent. A few studies (*n*=20 studies, 18.9%) explicitly reported funding disclosure statements, while most provided minimal or no related information. 18 studies (90.0%) appeared to report funding support from national sources (i.e. from one of the study countries), with the majority (*n*=15 studies, 99.2%) from Indian-based institutions. The formal recognition of TM and related practices by the Indian government (culminating in a dedicated central government-sponsored ministry of Ayush, formerly the Department of Indian Systems of Medicine and Homeopathy) in March 1995, and an integrated Ayurvedic Research Initiative in 2004 may somewhat explain the funder and researcher interests in TM-related research [11, 34]. Juxtaposing the funding details and publication timeline reveals a rather low rate of funding disclosures, indicative of a lack of governmental/international funding, which may partly explain the limited research in the topic area. The low funding disclosures is also suggestive of a funding gap in the prioritisation of TM research on zoonotic diseases from LMICs by international funding organisations.

### Synthesis of evidence on TM interventions for zoonotic disease conditions

#### Diversity of ethno-medicinal use of plant species

**Fig. 3:**
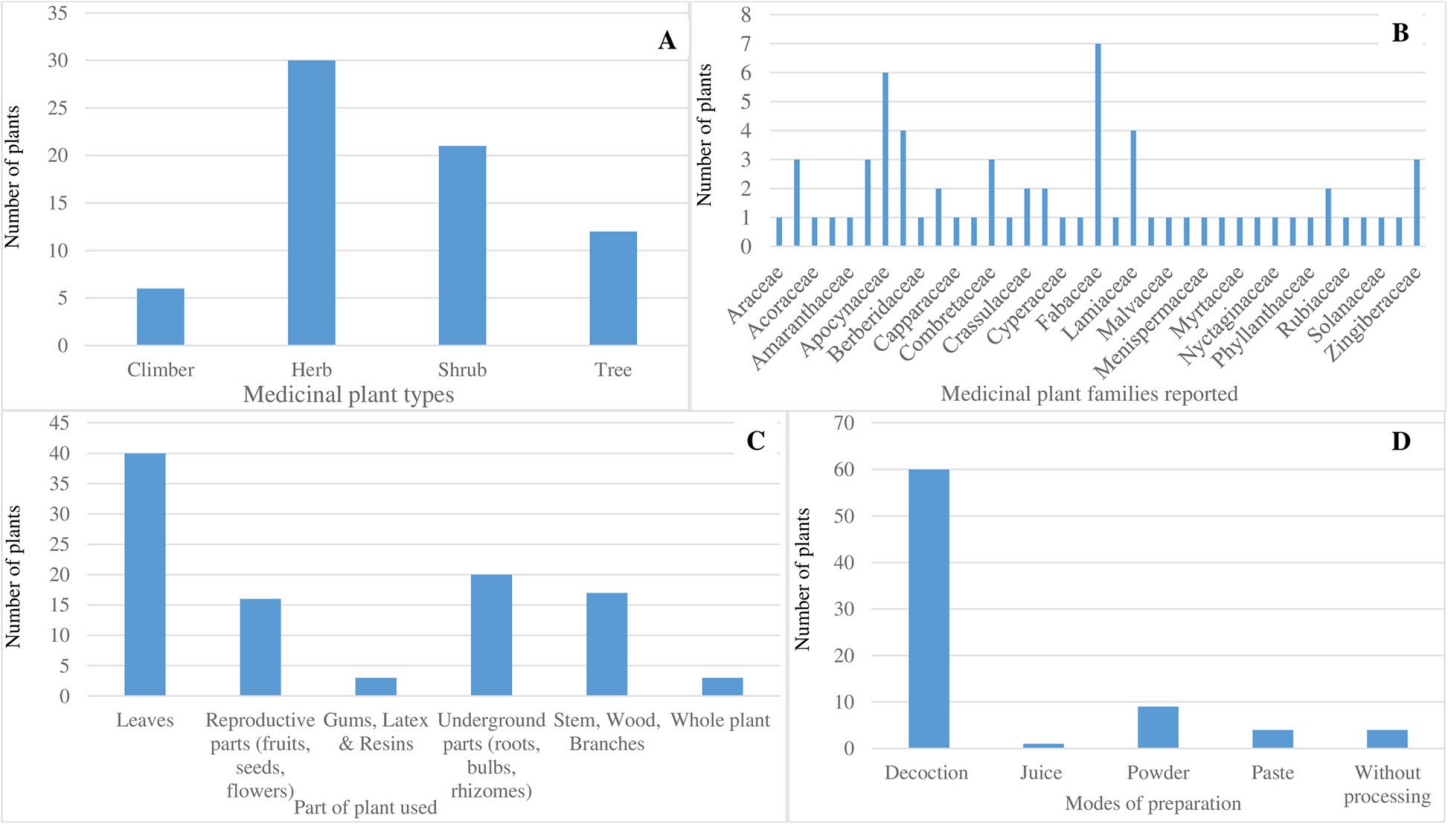
Medicinal plant characteristics and use for different zoonotic ailments. Bar charts shows the number of species by growth forms (**A**), category of plant species (**B**), parts of plant used (**C**) and specific modes of preparation reported (**D**)

Table 2 presents the ethno-medicinal uses of respective plant species as reported in the reviewed studies. We identified a total of 69 types of medicinal plant species belonging to 41 families recorded for treatment of 29 zoonotic disease conditions (Table 2). The medicinal plants studied have diverse growth forms including 30 herbs (43.5%), 21 shrubs (30.4%), 12 trees (17.4%) and 6 climbers (8.7%) (Fig. 3A). Of the diversity of plant families reported (*n*=41), Fabaceae was the largest family represented by 7 plants, followed by Apocynaceae with 6 plants, Asteraceae and Lamiaceae with 4 plants each, and Acanthaceae, Apiaceae, Combretaceae and Zingiberaceae with 3 plants each as evidenced in Fig. 3B. Of the plant-based remedies reported in reviewed studies, various parts of plants were used in the preparation of recipes to treat different ailments, with leaves being the most frequently used plant parts (*n*=40 plants) followed by the underground parts (i.e. roots, bulbs and rhizomes) (*n*=20 plants), stem, bark and branches (*n*=17 plants) and reproductive parts (i.e. fruits, seeds and flowers) (*n*=16 plants), whole plant and gums/plant sap (*n*=3 plants) respectively (Fig. 3C). On the mode of preparation of the plant-based remedies reported, decoction (*n*=60 plants) and powdered/ground (*n*=9 plants) are the most used. Decoction is an extraction method (i.e., an extraction method involving boiling herbal or plant material in water to dissolve the chemicals of the said material before administration. An oral route of administration was used for all the plant-based remedies, using a range of administration forms, including concoction (i.e. mixing water with an assortment of different plant parts) and powder (Table 2, Fig. 3D).

**Table 2:** Reported ethno-medicinal uses of plant species

**Scientific name**^**a**^	**Common name**	**Part used**	**Disease(s)**^**b**^	**Mode of preparation**	**Route of application (humans)**
Carica papaya	Pawpaw	Root and leaf	Dengue^5,6,7,89^	Decoction	Oral
Lasia spinosa	Lasia	Leaf	Trichinellosis^2,3^	Decoction	Oral
Acorus calamus	Sweet flag	Leaf	Leishmaniasis^3,4^	Powder and without processing	Oral
Boesenbergia rotunda	Fingerroot	Leaf	Leptospirosis^4,5^	Decoction	Oral
Rubia cordifolia	Indian madder	Root	Lymphatic filariasis^5,6^, dengue^5,6,7^, chikungunya^1,5,6^, shigellosis^83^, *Escherichia coli* infection^83^ and zika virus disease^5,6^	Decoction	Oral
Terminalia arjuna	Arjun tree	Bark	Dengue^5,6,7^	Decoction	Oral
Apium graveolens	Celery	Seed	Dengue^5,6,7^	Decoction	Oral
Psidium guajava	Common guava	Leaf and Bark	Avian influenza^10^, swine flu^10^, dengue^5,6,7,10^ and chikungunya^1,5,10^	Decoction	Oral
Achyranthes aspera Linn.	Apamarga	Leaf and seed	Rabies^11^	Paste	Topical
Abrus precatorius L	Rosary pea	Root, leaf and seed	Rabies^13^, tuberculosis^20^ and *Escherichia coli* infection^68^	Decoction and powder	Topical
Lawsonia inermis	Henna	Leaf	Cholera^15^	Decoction	Oral
Andrographis paniculata Nees	King of Bitters	Leaf	Dengue^5,6,7^ and viral hepatitis^17^	Decoction	Oral
Carissa spinarum	Conkerberry	Root	Helminthic infections^22^	Decoction	Oral
Tinospora cordifolia (Wild) Miers	Guduchi	Leaf, stem and root	Dengue^5,6,7,104^, leishmaniasis^3,8^ and swine influenza	Decoction	Oral
Azadirachta indica A	Neem tree	Root, leaf and stem bark and seed	Dengue^5,6,7^, chikungunya^1,5^, yellow fever, cholera^23,61^, echinococcosis^47^ and leishmaniasis^3,8,92^	Decoction, powder and without processing	Oral
Gynura angulosa DC (compositae)	Purple velvet plant	Leaf	Helminthic infections^24^	Decoction	Oral
Andrographis paniculata	Green chiretta	Leaf	Helminthic infections^27^	Decoction	Oral
Clerodendrum colebrookianum Walp	East Indian glory bower	Leaf	Helminthic infections^31^	Decoction	Oral
Ocimum basilicum	Holy Basil/ common basil	Root, leaf, flower and seed	*Escherichia coli* infection^57,86^, salmonellosis^57^, shigellosis^86^, swine flu^34^, leishmaniasis^3,8^, dengue^5,6,7,26,103,105^, chikungunya^1,5,14,15,103^ and yellow fever^105^	Decoction and paste	Oral and topical
Adenia gummifera	Snake-climber	Root	Lymphatic filariasis^5^	Decoction	Oral
Salvia officinales	Common garden sage	Leaf and stem	Covid-19^37^	Decoction	Oral
Kalanchoe pinnata	Life plant	Leaf	Leishmaniasis^3,41^ and *Escherichia coli* infection^52^	Decoction	Oral
Cordia alliodora	Spanish elm/Ecuador laurel/cypre/salmwood	Stem bark	*Escherichia coli* infection^43^	Decoction	Oral
Ficus benghalensis L.	Banyan	Leaf	Yellow fever and dengue^5^	Decoction	Oral
Ferula asafoetida	Devil’s dung	Latex	Cystic echinococcosis^48^	Dried latex and decoction	Oral
Anisomeles malabarica	Malabar catmint	Leaf and flower	Leishmaniasis^3^	Decoction	Oral
Momordica charantia	Bitter Gourd	Seed	*Escherichia coli* infection^55^, salmonellosis^55^ and shigellosis^55^	Decoction	Oral
Zingiber montanum	Cassumunar ginger	Rhizome	Cholera^59^	Decoction	Oral
Pongamia pinnata	Indian Beech	Leaf	*Escherichia coli* infection^60^, salmonellosis^60^, shigellosis^60^ and campylobacter jejuni^60^	Decoction	Oral
Strychnos nux-vomica	Strychnine tree	Bark and seed	Cholera^63^	Decoction and paste	Oral
Juglans regia	Walnut	Bark	Lymphatic filariasis^5,67^, onchocerciasis^67^ and schistosomiasis^67^	Decoction	Oral
Casuarina equisetifolia	Coastal she-oak	Leaf	Salmonellosis^69^	Decoction	Oral
Albizzia lebbeck	East Indian walnut	Leaf and bark	Salmonellosis^70^, shigellosis^70^ and *Escherichia coli* infection^70^	Decoction	Oral
Crateva nurvala	Barna and varuna	Stem bark	Rabies^71^	Decoction	Oral
Withania somnifera	Winter cherry	Root, leaf and seed	*Escherichia coli* infection^72^	Decoction	Oral
Terminalia chebula	Chebulic myrobalan	Seed	Salmonellosis^73^, shigellosis^95^ and *Escherichia coli* infection^95^	Decoction and powder	Oral
Terminalia arjuna	Arjun tree	Bark	Salmonellosis^76^ and *Escherichia coli* infection	Decoction	Oral
Ecbolium viride	Blue fox tail	Root	Dengue^5,6,7^, chikungunya^1,5^ and Zika virus disease^5^	Decoction	Oral
Artemisia absinthium	Absinth wormwood	Root	Dengue^5,6,7,83^ and lymphatic filariasis^5^	Decoction	Oral
Hippophae	Sea buckthorn	Leaf and seed	Herpes virus^82^	Decoction	Oral
Vitex nugundo	Chaste tree	Leaf and flower	Cholera^84^	Decoction	Oral
Tagetes erecta	African marigold	Flower and leaves	Yellow fever, dengue^5,6,7,88^ and lymphatic filariasis^88^	Decoction	Oral
Bryophyllum pinnatum	Air plant	Leaf	*Escherichia coli* infection^91^ and shigellosis^91^	Decoction and juice	Oral
Trichosanthes dioica Roxb.	Parwal	Root	Leishmaniasis^3,8^	Decoction	Oral
Hibiscus rosa-sinensis	Chinese hibiscus	Leaf	Helminthic infections^101^	Powder	Oral
Alstonia scholaris	Blackboard tree	Leaf, bark and latex	Leishmaniasis^3,8,66^ and lymphatic filariasis^5,35,46^	Decoction	Oral
Antidesmadiandrum Roth.	Sour currant shrub	Leaf	Rabies^10^	Decoction	Oral
Calotropis procera	Apple of Sodom	Root, leaf, latex and flower	Lymphatic filariasis^5^	Powder	Oral
Saraca indica	Asoka tree	Bark	Cholera^16^	Decoction	Oral
Boerhaavia diffusa Linn	Punarnava	Whole plant	Dengue^5,6,7,23^	Herbal syrup	Oral
Albizzia chinensis	Chinese albizzia	Bark	Scabies^70^	Decoction	Oral
Dalbergia sissoo Roxb.	North Indian rosewood	Leaf and whole plant	Anthrax^29^, foot and mouth disease^29^	Paste and without processing	Oral and topical
Coriandrum sativum	Cilantro or Chinese parsley	Leaf	Dengue^5,6,7,40^	Decoction	Oral
Cordia cylindrostachya	String bush/Scorpion weed/Black sage	Leaf	*Escherichia coli* infection^43^	Decoction	Oral
Agele marmelos	Bael	Root, leaf and flower	Cholera^96^	Decoction	Oral
Gloriosa superba	Glory lily	Leaf	Leishmaniasis^3,8,50^	Decoction	Oral
Cyperus rotundus	Purple nutsedge	Rhizome	*Escherichia coli* infection^53^ and cholera^53^	Decoction	Oral
Physalis minima Linn.	Country gooseberry	Root	Cholera^64^	Herbal syrup	Oral
Cassia siamea	Siamese cassia/ Kassod tree/cassod tree/ Cassia tree	Leaf	Lymphatic filariasis^5,65,83^, leishmaniasis^3,8,83^ and ascariasis^83^	Decoction	Oral
Cichorium intybus	Chicory	Root	Dengue^5,6,81^ and lymphatic filariasis^5,81^	Decoction	Oral
Zingiber officina	Ginger	Rhizome	*Escherichia coli* infection^85,99^, salmonellosis^85,99^ and shigellosis^85^	Decoction and without processing	Oral
Rose (Rosa spp.)	Cabbage rose	Flower	Yellow fever^88^, dengue^5,88^ and lymphatic filariasis^5,88^	Decoction and powder	Oral
Prunus cirasoidus	Himalayan wild cherry	Stem	Dengue^5,6,90^	Decoction	Oral
Catharanthus roseus	Madagascar periwinkle	Leaf	Dengue^5,6,105^, chikungunya^1,5,105^ and yellow fever^105^	Decoction	Oral
Myrica cerifera	Bayberry Candleberry	Root	Covid-19^106^	Decoction	Oral
Berberis aristata	Citra	Leaf	Leishmaniasis^3,8^	Decoction	Oral
Alium sativum	Garlic	Whole plant	*Escherichia coli* infection^85^, covid-19^100^	Decoction	Oral
Calotropis gigantean	Crown flower	Leaf and bark	Lymphatic filariasis^5^	Powder	Oral

^a^Plant species: Climber (  ), Herb (  ), Shrub (  ), Tree (  )

^b^References: 1=Mishra et al. (2011), 2=Yadav & Temjenmongla (2011), 3=Sidana & Farooq (2015), 4= Chander et al. (2016), 5=Gandhi et al. (2016), 6= Tamilventhan & Jayaprakash (2019), 7= Kumar et al. (2014), 8= Uddin et al. (2012), 9= Sharma et al. (2021), 10= Ahirwar et al. (2013), 11= Betlu (2013), 12= Mahawar & Jaroli (2007), 13= Bhatia et al. (2014), 14= Raghavendhar et al. (2019), 15= Sharma et al. (2009), 16= Jaroli et al. (2010), 17= Raval & Raval (2016), 18= Teronpi et al. (2012), 19= Singh et al. (2005), 20= Bhatia et al. (2014), 21= Harwansh et al. (2010), 22=Bharati & Sinha (2012), 23= Thakurta et al. (2007), 24= Yadav et al. (2014), 25= Meena et al. (2010), 26= Shankar et al. (2016), 27= Rahmatullah et al. (2013), 28=Banerjee et al. (2018), 29=Mishra et al. (2015), 30= Roy et al. (2016), 31=Yadav & Temjenmongla (2012), 32=Nath & Yadav (2016), 33=Yadav & Tangpu (2012), 34=Devi et al. (2018), 35=Choudhari (2018), 36=Vijaya & Yadav (2014), 37=Rao et al. (2020), 38= Niraj & Varsha (2020), 39=Ghosh et al. (2020), 40=Singh et al. (2016), 41=Ullah et al. (2016), 42=Raja et al. (2018), 43= Ozaa & Kulkarnia (2017), 44=Govindarajan et al. (2011), 45= Saxena et al. (2016), 46=Murthy et al. (2010), 47=Verma et al. (2013), 48=Moudgil et al. (2020), 49=Palbag et al. (2016), 50=Zahir et al. (2012), 51= Amutha et al. (2019), 52=Rahaman (2011), 53=Sonawane et al. (2017), 54= Manojja et al. (2019), 55=Das et al. (2015), 56=John et al. (2014), 57=Jayati et al. (2013), 58=Prasad et al. (2010), 59=Pattanaik et al. (2006), 60=Brijesh et al. (2006), 61=Tyagi et al. (2016), 62=Sharma et al. (2019), 63=Kushwaha et al. (2014), 64=Bora et al. (2016), 65=Kumar et al. (2014), 66=Singh et al. (2010), 67=Kale et al. (2011), 68=Bhatia et al. (2014), 69=Desai & Desai (2015), 70=Padamanabhanathy & Evanjelene (2013), 71=Bhattacharjee et al. (2012), 72=Srivastav & Das (2014), 73=Khan (2009), 74=Singh et al. (2020), 75=Manohar (2022), 76=Panda et al. (2011), 77=Venkateswarlu (2016), 78= Singh & Sharma (2013), 79=Anand & Lal (2016), 80=Appadurai et al. (2015), 81=Ali et al. (2018), 82=Patil &Chaudhary (2016), 83=Singh et al. (2020), 84=Alagesaboopathi (2009), 85=Divyesh et al. (2013), 86= Kalaivani et al. (2012), 87=Mariselvam et al. (2014), 88=Hajra et al. (2015), 89=Yadav et al. (2018), 90=Ghanshyam et al. (2018), 91= Sadhana et al. (2017), 92=Chouhan et al. (2015), 93=Arawwawalaand & Wickramaarachchi (2012), 94=Bhattacharya et al. (2013), 95=Mekala & KrishnaMurthy (2020), 96= Suja et al. (2017), 97=Kaur (2017), 98=Shobi et al. (2018), 99=Ambrin et al. (2020), 100=Kaus & Singh (2020), 101= Nath & Yadav (2015), 102=Ramalingam et al. (2018), 103=Uniyal et al. (2014), 104=Paul et al. (2021), 105=Gupta et al. (2020), 106=Singh et al. (2020)

**Fig. 4:**
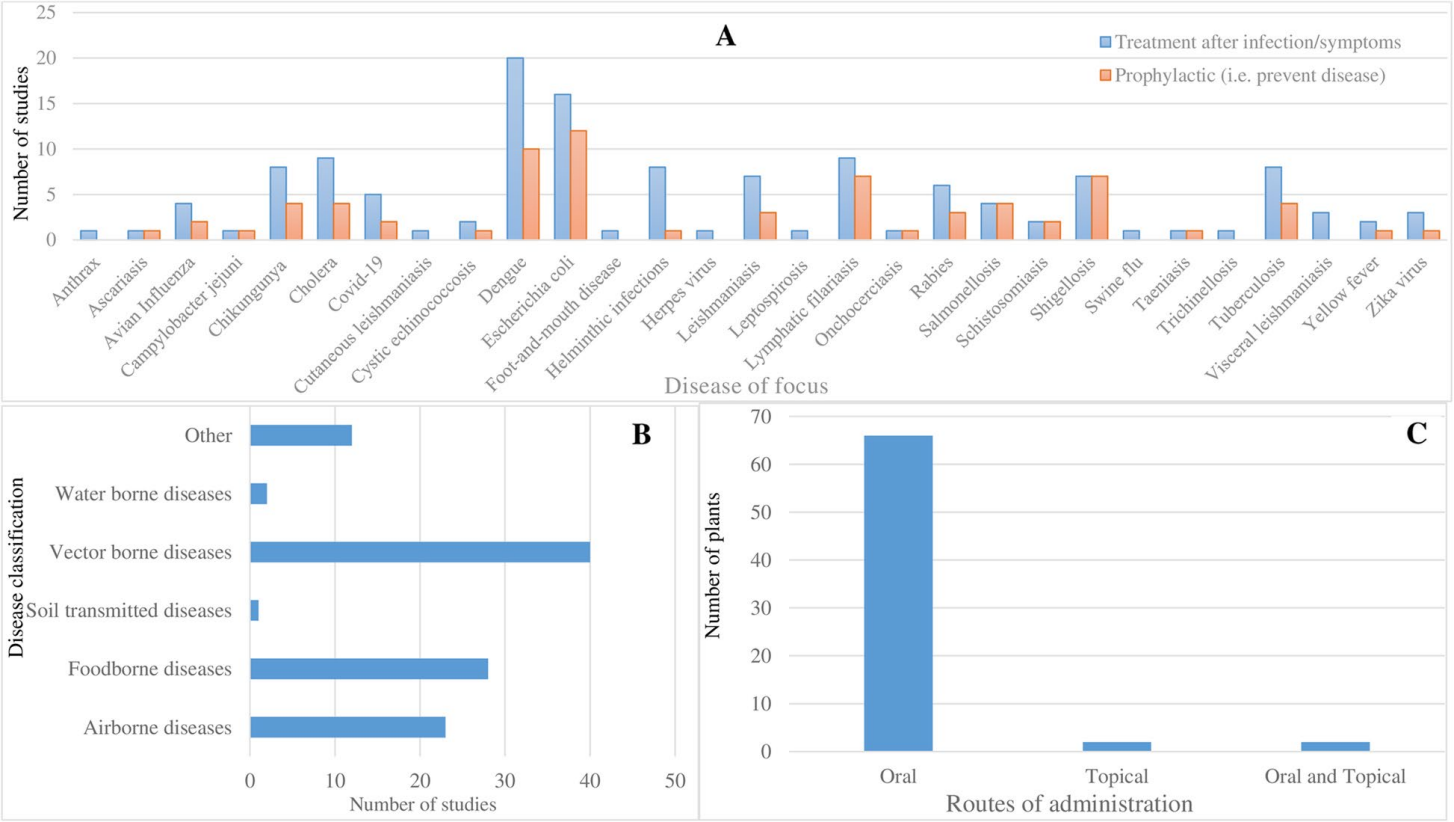
Reported disease conditions treated by plant-based remedies. Bar charts shows the number of papers by specific zoonotic diseases identified as being treated and/or prevented by reported TMs (**A**), disease transmission routes (**B**) and route of administration (**C**)

All 29 zoonotic diseases reportedly cured with medicinal plants are categorised into 5 transmission categories: in which vector-borne diseases are the most mentioned (with high number of studies, *n*=40 studies) followed by foodborne (*n*=28 studies) and airborne diseases (*n*=23 studies, respectively) (Fig. 4). As evidenced in Fig. 4A, most reviewed papers reported on dengue (*n*=20 studies), zoonotic *Escherichia coli* infection (*n*=16 studies), cholera and lymphatic filariasis (*n*=9 studies), chikungunya and tuberculosis (*n*=8 studies), leishmaniasis and shigellosis (*n*=7 studies) and rabies (*n*=6 studies) as the topmost zoonotic diseases reportedly treated by plant-based remedies. Nearly half of the reviewed studies (*n*=50 studies, 47.2%) reported on TMs that were utilised for both treatment and disease prevention (Fig. 4A). Of the 40 studies that reported on vector-borne infections, only 17.5% (*n*=7 studies) mentioned TMs for personal/animal protection against arthropod vectors, particularly dengue (*n*=6 studies) and lymphatic filariasis (*n*=1 study) [35–45]. A key observation in terms of TM research in the sub-region is the disproportionate focus on so-called priority zoonoses of concern (e.g. dengue, leishmaniasis, rabies), highlighting a knowledge gap on reported TM remedies for treating less visible, endemic and re-emerging zoonoses (e.g. scrub typhus and Kyasanur forest disease) affecting rural marginalised populations that have limited formal healthcare access and heavy dependence on traditional medicine remedies [26].

#### Diversity of zoo-therapeutic use of animal species

**Fig. 5:**
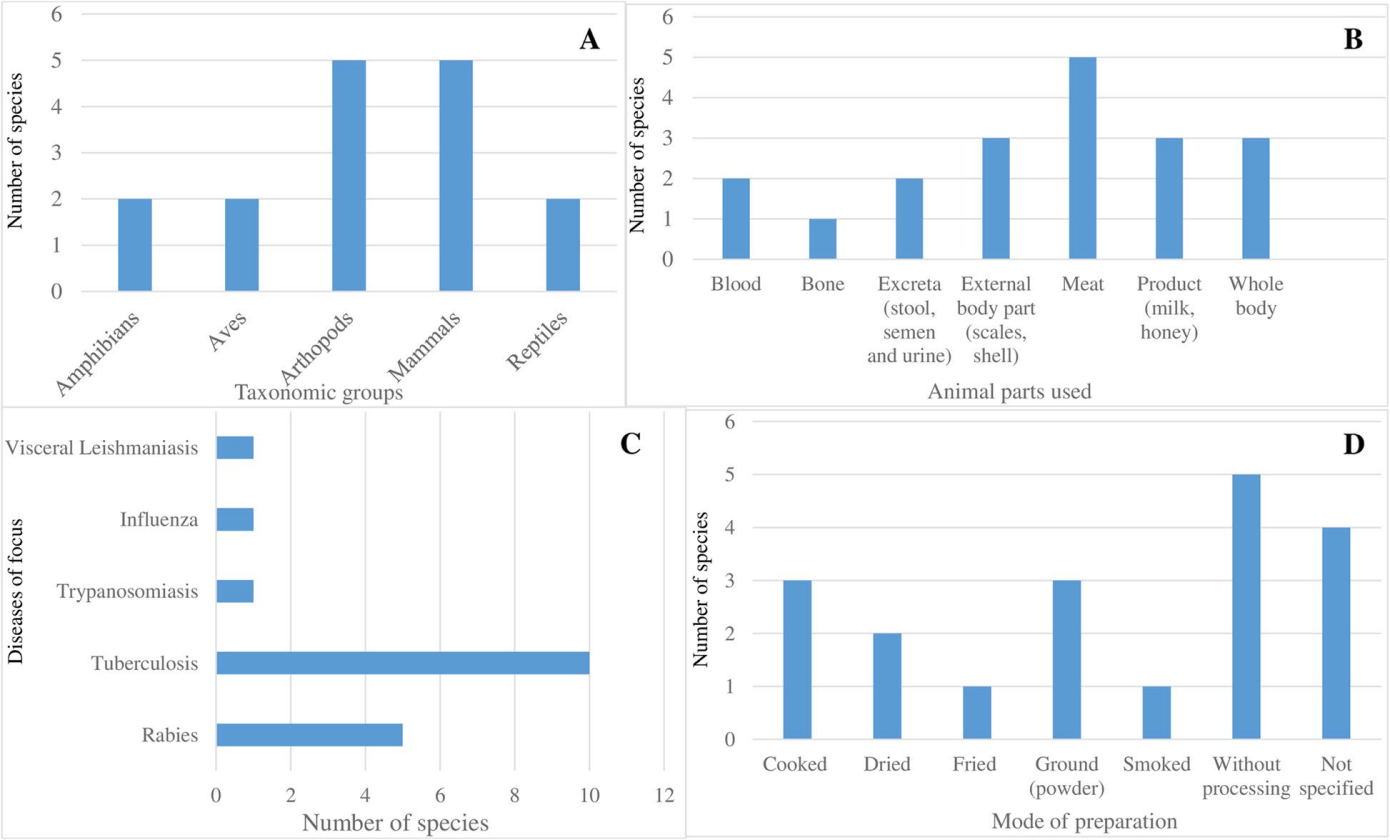
Characteristics of animal-based remedies used for different zoonotic ailments. Bar charts shows the number of species by taxonomic categories (**A**), animal parts used (**B**), specific zoonotic diseases (**C**) and modes of preparation reported (**D**)

As shown in Table 3, only 5 studies (representing 4.7% of the reviewed papers) reported on zoo-therapeutics (i.e. animal-derived medicinal resources) [28, 30, 42–44]. The described interventions were used in the treatment of 5 ailments and involved 16 different vertebrate (*n*=11, 68.8%) and invertebrate species (*n*=5, 31.2%) belonging to 6 taxonomic families. Mammals and arthropods occupied the highest uses (*n*=5 species apiece), followed by amphibians, birds and reptiles (*n*=2 species apiece) (Fig. 5A). Inferring from Fig. 5B, meat was the most extensively reported medicinal parts of the animals used (*n*=5 species), followed by whole body and external body parts (*n*=3 species), and blood and excreta (*n*=2 species) respectively. Of the reported animal species used as medicinal resources, 9 are included in the IUCN Red Data list [45]. Species such as Indian pangolin are listed as endangered while Common tortoise and the Indian rock python are listed as vulnerable and near threatened respectively in IUCN Red Data list (see Table 3). This highlights a seeming tension around the illegality of using protected animal species and their therapeutic potential for the maintenance of human health, particularly in remote settings. A further cross-tabulation analysis of the taxonomic classification and reported diseases showed that tuberculosis (66.7%) and rabies (16.7%) were the topmost diseases treated with the identified animal-based medicines.

**Table 3:** Reported zoo-therapeutic use of animal species

**Animal group**	**Family**	**Scientific name**	**Common name**^**a**^	**Animal part/product used**	**Disease(s)**^**b**^	**Mode of preparation**	**Mode of administration**
Amphibians	Dicroglossidae	Hoplobatrachus tigerinus	Indian bullfrog^**LC**^	Meat	Tuberculosis^16^	Cooked meat consumed	Oral
Amphibians	Geoemydidae	Kachuga tentoria	Indian tent turtle^**LC**^	Meat	Tuberculosis^11^	Cooked meat consumed	Oral
Aves	Anatidae	n/a	Duck	Meat	Tuberculosis^42^	Cooked meat consumed	Oral
Arthropods	Cancridae	Cancer pagurus	Edible crab^**LC**^	Shell	Tuberculosis^12^	Ash of Crab (ground)	Oral
Arthropods	Apoidea	Apis mellifera	Honeybee	Honey	Tuberculosis^42^	Raw honey consumed	Oral
Arthropods	Meloidae	Periplaneta americana	Cockroach	Whole body	Tuberculosis^11^	Dried	Oral
Arthropods	Meloidae	Monopterus cuchia	Gangetic mudeel^**LC**^	Whole body and blood	Visceral Leishmaniasis^18^	Raw blood consumed	Oral
Arthropods	Palaemonidae	Macrobrachium malcolmsonii	Indian river prawn^**LC**^	Whole body	Tuberculosis^12^	Dried	Oral
Mammals	Bovidae	Bos taurus	Ox	Dung	Mosquito repellent^42^	Smoked	Smoked
				Milk	Rabies and tuberculosis^42^	Raw milk consumed	Oral
Mammals	Cercopithecidae	Macaca sp.	Stump-tailed macaque^**VU**^	Blood	Tuberculosis^42^	Raw blood consumed	Oral
Mammals	Bovidae	Capra aegagrus hircus	Goat	Milk	Tuberculosis^42^	Raw milk consumed	Oral
Mammals	Equidae	Equus caballus	Horse	Semen	Rabies^12^	Not specified	Oral
Mammals	Manidae	Manis crassicaudata	Indian pangolin^**EN**^	Scales	Tuberculosis^11^	Dried scale crushed to powder and dissolved water	Oral
Reptiles	Pythonidae	Python molurus	Indian rock python^NT^	Bone	Rabies^42^	Not specified	Tying and banding
				Meat	Rabies^42^	Not specified	Oral and topical
Reptiles	Testudinidae	Testudo graeca	Common tortoise^**VU**^	Shell	Trypanosomiasis^42^	Not specified	Fumigation

^a^Conservation status of animal species (IUCN red list 2022): (EN= Endangered; LC= Least Concern, NT= Nearly Threatened, VU= Vulnerable);

^b^References: 1=Mishra et al. (2011), 2=Yadav & Temjenmongla (2011), 3=Sidana & Farooq (2015), 4= Chander et al. (2016), 5=Gandhi et al. (2016), 6= Tamilventhan & Jayaprakash (2019), 7= Kumar et al. (2014), 8= Uddin et al. (2012), 9= Sharma et al. (2021), 10= Ahirwar et al. (2013), 11= Betlu (2013), 12= Mahawar & Jaroli (2007), 13= Bhatia et al. (2014), 14= Raghavendhar et al. (2019), 15= Sharma et al. (2009), 16= Jaroli et al. (2010), 17= Raval & Raval (2016), 18= Teronpi et al. (2012), 19= Singh et al. (2005), 20= Bhatia et al. (2014), 21= Harwansh et al. (2010), 22=Bharati & Sinha (2012), 23= Thakurta et al. (2007), 24= Yadav et al. (2014), 25= Meena et al. (2010), 26= Shankar et al. (2016), 27= Rahmatullah et al. (2013), 28=Banerjee et al. (2018), 29=Mishra et al. (2015), 30= Roy et al. (2016), 31=Yadav & Temjenmongla (2012), 32=Nath & Yadav (2016), 33=Yadav & Tangpu (2012), 34=Devi et al. (2018), 35=Choudhari (2018), 36=Vijaya & Yadav (2014), 37=Rao et al. (2020), 38= Niraj & Varsha (2020), 39=Ghosh et al. (2020), 40=Singh et al. (2016), 41=Ullah et al. (2016), 42=Raja et al. (2018), 43= Ozaa & Kulkarnia (2017), 44=Govindarajan et al. (2011), 45= Saxena et al. (2016), 46=Murthy et al. (2010), 47=Verma et al. (2013), 48=Moudgil et al. (2020), 49=Palbag et al. (2016), 50=Zahir et al. (2012), 51= Amutha et al. (2019), 52=Rahaman (2011), 53=Sonawane et al. (2017), 54= Manojja et al. (2019), 55=Das et al. (2015), 56=John et al. (2014), 57=Jayati et al. (2013), 58=Prasad et al. (2010), 59=Pattanaik et al. (2006), 60=Brijesh et al. (2006), 61=Tyagi et al. (2016), 62=Sharma et al. (2019), 63=Kushwaha et al. (2014), 64=Bora et al. (2016), 65=Kumar et al. (2014), 66=Singh et al. (2010), 67=Kale et al. (2011), 68=Bhatia et al. (2014), 69=Desai & Desai (2015), 70=Padamanabhanathy & Evanjelene (2013), 71=Bhattacharjee et al. (2012), 72=Srivastav & Das (2014), 73=Khan (2009), 74=Singh et al. (2020), 75=Manohar (2022), 76=Panda et al. (2011), 77=Venkateswarlu (2016), 78= Singh & Sharma (2013), 79=Anand & Lal (2016), 80=Appadurai et al. (2015), 81=Ali et al. (2018), 82=Patil &Chaudhary (2016), 83=Singh et al. (2020), 84=Alagesaboopathi (2009), 85=Divyesh et al. (2013), 86= Kalaivani et al. (2012), 87=Mariselvam et al. (2014), 88=Hajra et al. (2015), 89=Yadav et al. (2018), 90=Ghanshyam et al. (2018), 91= Sadhana et al. (2017), 92=Chouhan et al. (2015), 93=Arawwawalaand & Wickramaarachchi (2012), 94=Bhattacharya et al. (2013), 95=Mekala & KrishnaMurthy (2020), 96= Suja et al. (2017), 97=Kaur (2017), 98=Shobi et al. (2018), 99=Ambrin et al. (2020), 100=Kaus & Singh (2020), 101= Nath & Yadav (2015), 102=Ramalingam et al. (2018), 103=Uniyal et al. (2014), 104=Paul et al. (2021), 105=Gupta et al. (2020), 106=Singh et al. (2020)

### Effects of TM interventions

As the effectiveness of TM interventions is critical to inform evidence-based policymaking, it was instructive to identify the range of interventions appraised in the reviewed studies. Effectiveness in this context refers to the reduction in symptom severity and/or mortality rate in infected people. Of the 106 reviewed studies, only 38 articles (35.8%) reported effectiveness (perceived or actual) of reported TM remedies against zoonotic diseases. Out of the 38 articles, 78.9% (*n*=30) were primary studies drawing mostly on quantitative cross-sectional data (*n*=27 studies, 71.1%) collated across different affected populations in different geographical contexts.

**Fig. 6:**
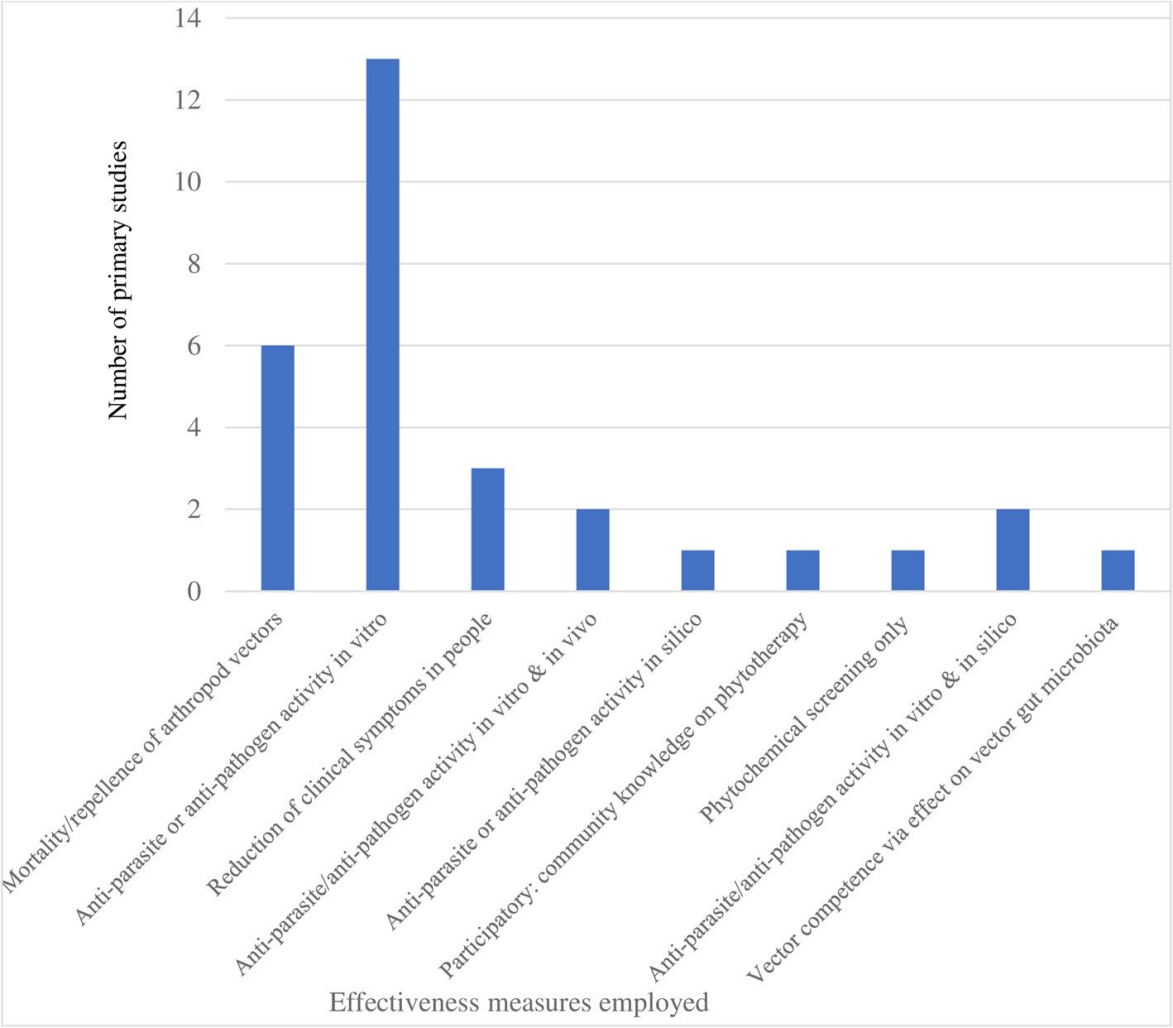
Measures used to assess effectiveness of traditional medicine interventions reported in respective studies

Concerning the 30 primary (i.e. not review) studies that measured some aspect of the effectiveness of plant products, 6 measured effects on mortality or repellency to arthropod disease vectors, 1 measured effects on adult vector gut microbiota (assumed to affect vector competence), 2 were purely in silico screening of phytochemical compounds for anti-pathogen properties (Fig. 6). Of the 19 studies that measured anti-pathogen activity of plant compounds (including anti-bacteria, anti-viral, anti-helminth and anti-parasite studies), 2 did so solely via in silico screening, 11 did solely via in vitro screening, and 5 employed in vivo screening in mouse, rat or shrimp models (2 of which included in vitro screening as well and 2 of which included in silico screening as well). Two of the in vivo studies in small mammal models looked beyond anti-pathogen activity at the ability of the compounds to inhibit haemorrhaging and secretion or to modulate the immune reaction to infection. Only two studies (6.6%) measured effects of plant compounds on reduction in symptoms of treated human patients (one of which was a case report on one patient, and one was a study involving 55 patients). One study measured perceived effectiveness of products by the community using participatory approaches. This indicates a picture of relatively few plant medicinal products or compounds progressing in testing beyond phytochemistry and in silico or in vitro screening to testing of efficacy in animals or humans (unless these tests are conducted in governmental institutes such as NITM but not published in the peer-reviewed literature).

There was limited or no information in terms of the perceptions of specific populations who utilised the reported TM interventions for the treatment of zoonotic diseases (except [42, 46–50]). This underscores the importance of high-quality randomised control trials and longitudinal evaluation studies to critically appraise the effectiveness of reported TM interventions for treatment of different zoonotic diseases. On the question of adverse/side effects (perceived or actual) associated with identified TM interventions, very few studies (*n*=5 studies) reported adverse effects (i.e. included reporting of them in the study protocol, but no adverse effects were found) which highlights an important lacuna in the extant literature on the sub-region [28, 43, 51–53]. These studies were mostly qualitative and characterised the perceptions of identified knowledge holders on TM remedies for treating specific zoonotic diseases, reporting none to mild side effects associated with identified TM remedies. For instance, Teronpi et al. [43] in their study of ichthyofauna usage in the treatment of tuberculosis among the Karbis of Assam (northern India) reported that patients suffering from goitre or leprosy (as co-occurring conditions with the TB) were barred from eating scaled fishes as they aggravated the disease condition.

## Discussion

### Scope of the literature and identified gaps

Amidst the long history of TM practice and growing appreciation its importance as alternative therapeutic treatment [11, 23], it is critical to better understand the characteristics of TM interventions and related disease conditions and scope out evidence gaps and opportunities to inform future research and practices**.** In this review, we examined the literature for documented TM interventions against zoonotic diseases on the Indian sub-continent and found evidence of varied applications for the treatment and prevention of numerous zoonoses.

While our findings broadly corroborate prior evidence on importance of TM for reducing the burden of zoonotic diseases in the sub-region [29, 54] and worldwide [9, 23], the limited sample of 106 studies we identified suggest that there is much to learn about TM interventions for controlling zoonotic diseases beyond the scientific literature. Evidence on their effectiveness and clinical safety is particularly lacking, with only 38 of reviewed studies reporting on effectiveness and adverse effects of TM application to specific disease conditions respectively. Of the 38 studies, a wide range of different metrics for effectiveness of TMs are used, drawn from disparate fields and the perceptions of the affected communities are often neglected in these methodologies. This highlights the need for long-term systematic studies to investigate the effectiveness of interventions and sustainability of TM interventions for zoonotic diseases utilising a range of inter-disciplinary methods and metrics. Only 9 studies were clinical studies and these were conducted between 2006 and 2020 [5, 43, 55–60]. Most of the studies were single-site observational studies or qualitative studies with limited inference on effectiveness. Besides, the limited evidence base perhaps also highlights the importance of recourse to the non-English Ayurvedic and Unani literature databases (which due to language limitation were excluded in our literature search) that might hold relevant studies/ reports beyond the English international peer-reviewed literature database. The limited evidence also highlights the limits of the scientific literature in providing a comprehensive view of how widespread and well utilised TMs actually are, particularly in rural contexts.

Concerning study classification, with a few exceptions (such as studies reporting zoo-therapeutic-based interventions) most of the TM remedies reported were ethno-medicinal/ plant-based. Considering that about 25% of contemporary drugs are derived from plants, the predominance of studies reporting on ethno-medicinal based remedies for zoonotic infections is not surprising [54, 61]. In any event, this lends further credence to observations about vast potential of plant-derived products as therapies for different emerging disease conditions, highlighting the importance of in-situ conservation of medicinal plants to serve as genetic pools (gene banks) for future ethno-pharmacological research and development [30]. Moreover, the utilisation of some wild endangered faunal species in the TM remedies (e.g. Indian pangolin, Common tortoise) also raises grave conservation and sustainability concerns in light of existing wildlife protection legislation in the focal countries and highlights the need for careful evaluation of these costs compared to the verified benefits of utilising a TM treatment. For instance, section 9 of the Indian Wildlife (Protection) Act 1972 strictly prohibits the hunting of any wild animal specified in schedule I, II, III and IV except as provided under section 11 and section 12. From an ethno-zoological perspective, the use of animal-based medicines, particularly those derived from wild endangered fauna species, have far-reaching negative implications for sustainable biodiversity management. This underscores the importance of systematic documentation and reporting of traditional knowledge of animal-based medicines for informing future biodiversity conservation policy [5, 42]. We further found that researchers tended towards the study of environmentally mediated disease topics, particularly those that are vector-borne and high profile (e.g., dengue, lymphatic filariasis and chikungunya), suggestive of a publication bias in favour of vector-borne studies relative to foodborne or airborne zoonoses in the international peer-reviewed journals. This notwithstanding, there is very limited evidence on the effectiveness of TM interventions for personal and/or animal protection from vectors to reduce disease/pathogen exposure in the studied region. This is despite evidence from household surveys demonstrating fairly widespread use of homemade tick repellents for humans and animals for forest-dependent communities affected by tick-borne zoonoses in India [26].

Interestingly, the extant traditional medicine-zoonoses research involved very limited international collaborations (i.e. scholars whose reported institutional affiliations fall outside of the Indian subcontinent) suggestive of the predominance of in-country scholar authored publications in the academic and clinical knowledge base. To the extent that there are government institutes devoted to TM that can operate without the need for external research funding (e.g. India’s NITM) may have contributed to this development. From a broader research/knowledge equity perspective, this is a positive and encouraging development which underscores a window of opportunity for shoring up strategic funding investment towards bridging the longstanding scientific ‘knowledge production’ gap between global North and South researchers and strengthening capacity in this sub-field. We further note that there exists marked difference in research foci in the sub-region, with disproportionate TM research reporting on India. This narrow focus of the extant scholarship highlights a reporting bias skewed towards India, potentially occasioned by oversampling and/or predominance of relevant studies published in Indian peer-reviewed journals. This bias is perhaps further compounded by the exclusion of non-English publications and/or ‘obscure’ and inaccessible in-country databases. Whereas there is geographical similarity across the sub-region, the marked differences in health system infrastructure and capacity as well as the variation in local policy and TM practice has meant that the findings may not be generalisable to all the studied countries. Understanding the aggregate picture of TM research and practice focussed on zoonotic diseases across the sub-region, in addition to nuanced country-specific observations particularly in the underrepresented countries, is necessary for generalisation of the evidence base, and potentially for knowledge sharing among affected communities, and should be a key area of future research.

### Limitations

Despite the long span and breadth of the peer-reviewed evidence collated which afforded the opportunity to identify and evaluate research gaps, here are still limitations in our study design, implementation and scope worth acknowledging. For practical reasons, we only included studies reported in English which imply that relevant studies reported in local languages might have been missed. Further, our assessment focussed on academic peer-reviewed literature, implying that we only deduce from TM remedies described in the scientific literature and not all those utilised in practice, particularly if they have yet to be published. There is evidence attesting to the non-disclosure of some TM remedies/knowledge held within communities (and passed between generations) due to bio-piracy and ethical concerns about documenting and sharing traditional knowledge more widely [52, 62]. Besides, some scholars have highlighted the reticence of local communities to describe TM practices, particularly those that might be deemed illegal and/or culturally sensitive in their socio-political and legal contexts [45, 54]. We thus acknowledge that there may be bias in communicating the ‘real’ TMs that are utilised and/or the reporting of common TM remedies, with those deemed to be effective more likely to be reported in the international peer-reviewed literature. Follow-up research exploring the questions of what information exist on TM remedies for treating zoonotic illnesses among different sub-population classes, the knowledge holders and geographical distribution of TM usage through key-informant interviews, focus group discussions and analysis of grey literature would be a useful complement to this study. While a key strength of this scoping review resides in its broad scope of the evidence (which to our knowledge is one of the first scoping assessment of TM interventions for zoonotic diseases in the sub-region), we acknowledge that our approach only lends itself to narrative synthesis limiting detailed and in-depth analysis required to assess effectiveness and uptake of respective TM interventions and their source quality. There is thus the need for more focussed systematic assessment on the clinical safety and effectiveness of inventoried TM interventions for treatment and prevention of zoonotic diseases across the subcontinent, leveraging mixed method approaches (i.e. qualitative and quantitative data gathering techniques). Notwithstanding these limitations, however, this scoping review provides insights into the scope and distribution of the evidence base on TM interventions for (re-)emerging zoonotic diseases, highlighting avenues for future research directions.

## Conclusion and way forward

This scoping review identified 106 articles pertaining to traditional medicine remedies for treating 29 zoonotic diseases across the Indian subcontinent context. Although there is strong evidence of TM’s growing popularity as alternative therapeutic treatment for different zoonotic ailments in the sub-region, very few studies reported on their effectiveness and associated side effects among different populations. The Indian subcontinent offers substantial opportunities to systematically study TM treatments for endemic and emerging zoonotic diseases for evidence-based policymaking in traditional medicine. Reflecting on future research directions, we thus present cross-cutting themes that can frame the future research and practice agenda on TM interventions for the control and prevention of zoonoses on the Indian subcontinent.

We first suggest intensification of research efforts on the clinical safety and efficacy of TM products and interventions to harness their full potential as alternative therapeutic treatment for zoonotic diseases. This is particularly important for those endemic zoonoses commonly reported as disproportionately impacting vulnerable rural communities that are often detached from formal healthcare systems. A promising approach in this regard could be locale and placebo analysis of TM interventions and usage by specific sub-populations evaluating dosage requirements, side-effects and cost effectiveness. To facilitate such granular scale assessments, reported data on costs and patient-reported/ health-related quality of life outcomes which are currently uncommon in TM evaluation research remain paramount. Besides, the standardisation of methodological protocols (e.g. high-quality randomised controlled trials commonly touted as the gold standard for clinical evaluative studies), and longitudinal evaluative studies remain necessary for robust comparative assessment of the safety and efficacy of commonly reported TM remedies for treatment of zoonotic diseases. In order to place the TM remedies on scientific platforms it is necessary to identify the bioactive molecules (which also helps in quality control) and generate knowledge on the mechanisms underlying their effectiveness. A positive development in the context of TM research in the sub-region is the widespread patronage of Ayurvedic and Unani practice vis-à-vis their formal recognition in national health policy frameworks (e.g. India’s ministry of Ayush and National Institute of Traditional Medicine) [11], which provides an incremental incentive to develop and strengthen comprehensive databases of TM therapies to inform clinical practice and interventions. Achieving this also requires a greater focus on knowledge co-production to help better align the traditional medicine-zoonoses scholarship to stakeholder priorities in the region.

The second point is that more ethno-zoological studies are needed to build up the limited evidence base on inventoried animal species used for treatment of different zoonotic disease conditions in the sub-region. In this sense, a systematic review of the grey literature and inventoried databases of the established entities viz. institutions under AYUSH, Centre for Scientific & Industrial Research (CSIR) (e.g. Traditional Knowledge Digital Library (TKDL), Indian Institute of Integrative Medicine (IIIM) and Central Institute of Medicinal and Aromatic Plants (CIMAP)), and the Indian Council for Medical Research (ICMR), is critical to comprehensively map the evidence base on traditional medicine knowledge on different animal species and their medicinal value. Due to time and access constraints, we could not undertake a full appraisal of the grey literature particularly, the inventoried databases of the government institutions which required negotiated access. Going forward, the “One Health” approach (which recognises the interconnectedness of human health, wildlife and domestic animal health and the environment and the value of evaluating these interactions in an integrated manner, [63–65]) provides a useful analytical entry point for future studies evaluating ethno-medicinal and zoo-therapeutic uses of flora and fauna species for the prevention and control of emerging zoonotic diseases, particularly across the tropics that are characterised by huge faunal and floral biodiversity.

A related point is the urgent need to encourage and sustain research on “One Health” focussed collaborations and expertise sharing across the sub-region. Given the marked difference and history between focal countries in terms of research and development infrastructure, strategic cross-country collaborations researching into TM remedies for common priority diseases of interest could contribute to turning the tide on the high burden of zoonotic diseases in the sub-region. Leveraging India’s enduring TM research capacity and infrastructure provides a good window of opportunity to advance such strategic collaborations to augment the ‘research deficit’ in other less established contexts in the sub-region. We nonetheless acknowledge that developing and sustaining such regional collaborative platforms relies on reconciling strategic political and economic priorities and interests, which often undergird critical decisions on cross-sectoral collaborations let alone cross-country collaborations.

Although there is evidence of support for TM research and development in the sub-region, we further highlight the need to increase research funding investment to incentivise the forging and strengthening collaborations and expertise sharing. A case in point is the Department of AYUSH, government of India which has dedicated funds allocated for research in Ayurveda, yoga, unani, siddha and homeopathy. Another positive indication is the recent agreement between the World Health Organisation (WHO) and Government of India to establish the global centre for traditional medicine (supported by an investment of USD 250 million from the latter) to harness the potential for traditional medicine worldwide through modern science and technology [66]. Such strategic funding investments provide an avenue to progressively accumulate empirical findings that support coherent theory building and inform policy decision-making on traditional medicine practice in the sub-region and globally. The coronavirus pandemic serves as stark reminder about the need for integrative solutions to effectively address the growing threat of emerging infectious diseases, particularly as a critical health ‘safety net’ for the majority of the rural populations in LMICs, with poor access to formal healthcare systems [1, 26].

### Supplementary Information

Supplementary Material 1. 

## Data Availability

The datasets generated from this study have been appended as supplementary information.

## Abbreviations

TM: Traditional medicine
LMICs: Low-and middle-income countries
WHO: World Health Organisation
