# Supplementary material for: What is the state of the art on traditional medicine interventions for zoonotic diseases in the Indian subcontinent? A scoping review of the peer-reviewed evidence base
Source: BMC Complement Med Ther. 2024 Jun 29;24:249. doi: 10.1186/s12906-024-04553-8 (PMC11218393; doi:10.1186/s12906-024-04553-8)
Supplement: Supplementary file 1 — Supplementary Material 1. [file 12906_2024_4553_MOESM1_ESM.docx]

Table S1. Sources included in the analysis

| **No.** | **Authors** | **DOI** | **Country** |
| --- | --- | --- | --- |
| 1 | Mishra et al. (2011) |  | India |
| 2 | Yadav & Temjenmongla (2011) | 10.1007/s00436-011-2551-9 | India |
| 3 | Sidana & Farooq (2015) | 10.3329/bjp.v10i2.22674 | India |
| 4 | Chander et al. (2016) | 10.1080/14786419.2015.1046068 | India |
| 5 | Gandhi et al. (2016) | 10.1007/s13744-016-0386-x | India |
| 6 | Tamilventhan & Jayaprakash (2019) | 0974-360X | India |
| 7 | Kumar et al. (2014) | 10.3389/fpubh.2014.00147 | India |
| 8 | Uddin et al. (2012) | 10.3109/14756366.2011.604853 | Pakistan |
| 9 | Sharma et al. (2021) | 10.1007/s13337-021-00685-4 | India |
| 10 | Ahirwar et al. (2013) |  | India |
| 11 | Betlu (2013) |  | India |
| 12 | Mahawar & Jaroli (2007) | 10.1186/1746-4269-3-25 | India |
| 13 | Bhatia et al. (2013) |  | India |
| 14 | Raghavendhar et al. (2019) | 10.1016/j.virol.2019.04.007 | India |
| 15 | Sharma et al. (2009) |  | India |
| 16 | Jaroli et al. (2010) |  | India |
| 17 | Raval & Raval (2016) |  | India |
| 18 | Teronpi et al. (2012) | 10.4103/0257-7941.118547 | India |
| 19 | Singh et al. (2005) | 10.1016/j.jep.2004.12.032 | India |
| 20 | Bhatia et al. (2013) | 10.1016/j.jep.2013.12.017 | India |
| 21 | Harwansh et al. (2010) |  | India |
| 22 | Bharati & Sinha (2012) |  | India |
| 23 | Thakurta et al. (2007) | 10.1016/j.jep.2007.01.022 | India |
| 24 | Yadav et al. (2014) | 10.1080/22311866.2014.939716 | India |
| 25 | Meena et al. (2010) |  | India |
| 26 | Shankar et al. (2016) | 10.20431/2455-7153.0301002 | India |
| 27 | Rahmatullah et al. (2013) | 10.1089/acm.2012.0227 | Bangladesh |
| 28 | Banerjee et al. (2018) | 10.1007/s13337-018-0465-1 | India |
| 29 | Mishra et al. (2015) |  | India |
| 30 | Roy et al. (2016) | 10.3109/13880209.2016.1139600 | India |
| 31 | Yadav & Temjenmongla (2012) | 10.1007/s00436-012-2908-8 | India |
| 32 | Nath & Yadav (2016) | 10.5455/jice.20160521124439 | India |
| 33 | Yadav & Tangpu (2012) | 10.1007/s00436-011-2596-9 | India |
| 34 | Devi et al. (2018) |  | India |
| 35 | Choudhari (2018) | 10.30732/ijbbb.20180303002 | India |
| 36 | Vijaya & Yadav (2014) | 10.1007/s12639-014-0560-1 | India |
| 37 | Rao et al. (2020) |  | India |
| 38 | Niraj & Varsha (2020) | 10.14719/pst.2020.7.3.831 | India |
| 39 | Ghosh et al. (2020) |  | India |
| 40 | Singh et al. (2016) |  | India |
| 41 | Ullah et al. (2016) |  | India |
| 42 | Raja et al. (2018) | 10.20959/wjpps201812-12750 | India |
| 43 | Ozaa & Kulkarnia (2017) |  | India |
| 44 | Govindarajan et al. (2011) |  | India |
| 45 | Saxena et al. (2016) | 10.1007/s13337-016—0307-y | India |
| 46 | Murthy et al. (2010) | 10.1055/s-0030-1250452 | India |
| 47 | Verma et al. (2013) | 10.1155/2013/308515 | India |
| 48 | Moudgil et al. (2020) | 10.24099/vet.arhiv.0502 | India |
| 49 | Palbag et al. (2016) |  | India |
| 50 | Zahir et al. (2012) | 10.1016/j.exppara.2012.06.012 | India |
| 51 | Amutha et al. (2019) |  | India |
| 52 | Rahaman (2011) | 10.5897/AJPP11.273 | Bangladesh |
| 53 | Sonawane et al. (2017) | 10.22377/ijgp.v11i01.856 | India |
| 54 | Manojj et al. (2019) | 10.1016/j.sajb.2019.09.021 | India |
| 55 | Das et al. (2015) |  | India |
| 56 | John et al. (2014) | 10.1016/j.sjbs.2014.09.009 | India |
| 57 | Jayati et al. (2013) |  | India |
| 58 | Prasad et al. (2010) | 10.4103/0974-8520.72410 | India |
| 59 | Pattanaik et al. (2006) | 10: 189-197.2006 | India |
| 60 | Brijesh et al. (2006) | 10.1631/jzus.2006.B0665 | India |
| 61 | Tyagi et al. (2016) |  | India |
| 62 | Sharma et al. (2019) | 10.22377/ijgp.v13i04.2703 | India |
| 63 | Kushwaha et al. (2014) |  | India |
| 64 | Bora et al. (2016) |  | India |
| 65 | Kumar et al. (2014) | 10.3389/fpubh.2014.00147 | India |
| 66 | Singh et al. (2010) | 10.1002/jat.1498 | India |
| 67 | Kale et al. (2011) |  | India |
| 68 | Bhatia et al. (2013) |  | India |
| 69 | Desai & Desai (2015) |  | India |
| 70 | Padamanabhanathy & Evanjelene (2013) |  | India |
| 71 | Bhattacharjee et al. (2012) | 10.1016/S2221-1691(12)60379-7 | India |
| 72 | Srivastav & Das (2014) |  | India |
| 73 | Khan (2009) |  | India |
| 74 | Singh et al. (2020) | 10.37398/JSR.2020.640106 | Indian sub-continent |
| 75 | Manohar (2022) | 10.4103/0257-7941.122994 | India |
| 76 | Panda et al. (2011) | 10.1080/22311866.2011.10719091 | India |
| 77 | Venkateswarlu (2016) |  | India |
| 78 | Singh & Sharma (2013) |  | India |
| 79 | Anand & Lal (2016) |  | India |
| 80 | Appadurai et al. (2015) | 10.1016/j.parepi.2016.03.004 | India |
| 81 | Ali et al. (2018) | [10.1016/j.parint.2018.08.005](https://doi.org/10.1016/j.parint.2018.08.005) | India |
| 82 | Patil &Chaudhary (2016) | International Journal of Green Pharmacy | India |
| 83 | Singh et al. (2020) |  | India |
| 84 | Alagesaboopathi (2009) |  | India |
| 85 | Divyesh et al. (2013) |  | India |
| 86 | Kalaivani et al. (2012) | 10.1080/22311866.2012.10719105 | India |
| 87 | Mariselvam et al. (2014) | 10.1016/j.saa.2014.03.066 | India |
| 88 | Hajra et al. (2015) | 10.1007/s12639-015-0719-4 | India |
| 89 | Yadav et al. (2018) | 10.20959/wjpr20207-17799 | India |
| 90 | Ghanshyam et al. (2018) |  | India |
| 91 | Sadhana et al. (2017) |  | Pakistan |
| 92 | Chouhan et al. (2015) |  | Pakistan |
| 93 | Arawwawalaand & Wickramaarachchi (2012) |  | Sri Lanka |
| 94 | Bhattacharya et al. (2013) | 10.4103%2F0974-8490.110540 | India |
| 95 | Mekala & KrishnaMurthy (2020) |  | India |
| 96 | Suja et al. (2017) | doi.org/10.24321/0019.5138.201732 | India |
| 97 | Kaur (2017) |  | India |
| 98 | Shobi et al. (2018) | 10.15406/jabb.2018.05.00123 | India |
| 99 | Ambrin et al. (2020) | doi.org/10.17582 | Pakistan |
| 100 | Kaus & Singh (2020) |  | India |
| 101 | Nath & Yadav (2015) |  | India |
| 102 | Ramalingam et al. (2018) | 10.4103%2Fayu.AYU_144_17 | India |
| 103 | Uniyal et al. (2014) |  | India |
| 104 | Paul et al. (2021) | 10.22271/phyto.2021.v10.i2f.13847 | India |
| 105 | Gupta et al. (2020) | 10.1007/s42690-021-00479-7 | India |
| 106 | Singh et al. (2020) |  | India |

Table S2. Characteristics of citations

| **No.** | **First author (year)** | **Journal** | **Country** | **Disease(s)** | **Study design** |
| --- | --- | --- | --- | --- | --- |
| 1 | Mishra et al. (2011) | Indian Journal of Pharmaceutical Sciences | India | Chikungunya | Experimental study |
| 2 | Yadav & Temjenmongla (2011) | Parasitology Research | India | Trichinellosis | Experimental study |
| 3 | Sidana & Farooq (2015) | Journal of the Bangladesh Pharmacological Society | India | Leishmaniasis | Experimental study |
| 4 | Chander et al. (2016) | Natural Product Research | India | Leptospirosis | Experimental study |
| 5 | Gandhi et al. (2016) | Neotropical Entomology | India | Lymphatic filariasis, dengue, chikungunya and Zika Virus | Experimental study |
| 6 | Tamilventhan & Jayaprakash (2019) | Research Journal of Pharmacy and Technology | India | Dengue | Experimental study |
| 7 | Kumar et al. (2014) | Frontiers in Public Health | India | Dengue | Experimental study |
| 8 | Uddin et al. (2012) | Journal of Enzyme Inhibition and Medicinal Chemistry | Pakistan | Cutaneous leishmaniasis | Experimental study |
| 9 | Sharma et al. (2021) |  | India | Chikungunya | Experimental study |
| 10 | Ahirwar et al. (2013) | International Journal of Science and Research | India | Rabies | Mixed-methods: survey and interviews |
| 11 | Betlu (2013) | Journal of Ethnobiology and Ethnomedicine | India | Tuberculosis | Interview |
| 12 | Mahawar & Jaroli (2007) | Journal of Ethnobiology and Ethnomedicine | India | Tuberculosis, rabies and herpes | Interview |
| 13 | Bhatia et al. (2013) | Journal of Ethnopharmacology | India | Rabies | Mixed-methods: survey, focus groups, and interviews |
| 14 | Raghavendhar et al. (2019) | Virology | India | Chikungunya | Experimental study |
| 15 | Sharma et al. (2009) | Indian Journal of Pharmacology | India | Cholera | Experimental study |
| 16 | Jaroli et al. (2010) | Journal of Ethnobiology and Ethnomedicine | India | Tuberculosis | Mixed-methods: survey and interviews |
| 17 | Raval & Raval (2016) | Ancient Science of Life | India | Viral hepatitis | Review |
| 18 | Teronpi et al. (2012) | Ancient Science of Life | India | Visceral leishmaniasis | Mixed-methods: survey and interviews |
| 19 | Singh et al. (2005) | Journal of Ethnopharmacology | India | Visceral leishmaniasis | Experimental study |
| 20 | Bhatia et al. (2013) | Indo American Journal of Pharmaceutical Research | India | Rabies and tuberculosis | Review |
| 21 | Harwansh et al. (2010) | International Journal of Pharmaceutical Sciences and Research | India | Helminth infections | Experimental study |
| 22 | Bharati & Sinha (2012) | International journal of Ayurvedic & Herbal Medicine | India | Dengue | Experimental study |
| 23 | Thakurta et al. (2007) | Journal of Ethnopharmacology | India | Cholera | Experimental study |
| 24 | Yadav et al. (2014) | Journal of Biologically Active Products from Nature | India | Helminthic infections | Experimental study |
| 25 | Meena et al. (2010) | International Journal of Contemporary Research and Review | India | Leishmaniasis | Review |
| 26 | Shankar et al. (2016) | 10.20431/2455-7153.0301002 | India | Dengue | Clinical study |
| 27 | Rahmatullah et al. (2013) | 10.1089/acm.2012.0227 | Bangladesh | Visceral leishmaniasis and tuberculosis | Ethnobotanical survey |
| 28 | Banerjee et al. (2018) | 10.1007/s13337-018-0465-1 | India | Chikungunya | Laboratory study |
| 29 | Mishra et al. (2015) |  | India | Anthrax | Review |
| 30 | Roy et al. (2016) | Pharmaceutical Biology | India | Zoonotic tapeworm Hymenolepis diminuta | Laboratory study |
| 31 | Yadav & Temjenmongla (2012) | Parasitology Research | India | intestinal | Experimental study |
| 32 | Nath & Yadav (2016) | Journal of Intercultural Ethnopharmacology | India | hymenolepis diminuta | Mixed-methods: survey and interviews |
| 33 | Yadav & Tangpu (2012) | Parasitology Research | India | Hymenolepis diminuta | Qualitative: interviews |
| 34 | Devi et al. (2018) | European Journal of Biomedical and Pharmaceutical sciences | India | Swine flu | Review |
| 35 | Choudhari (2018) | CSVTU International Journal of Biotechnology, Bioinformatics and Biomedical | India | Lymphatic filariasis | Review |
| 36 | Vijaya & Yadav (2014) | Journal of Parasitic Diseases | India | Hymenolepiasis | In vitro |
| 37 | Rao et al. (2020) | Indian Journal of Traditional Knowledge | India | Covid-19 | Review |
| 38 | Niraj & Varsha (2020) | Plant Science Today | India | Covid-19 | Review |
| 39 | Ghosh et al. (2020) | Journal of Ayurvedic and Herbal Medicine | India | Covid-19 | Review |
| 40 | Singh et al. (2016) | Journal of Chemical and Pharmaceutical Research | India | Dengue | Review |
| 41 | Ullah et al. (2016) | Phytotherapy Research | India | Leishmaniasis | Ethnobotanical survey |
| 42 | Raja et al. (2018) | World Journal of Pharmacy and Pharmaceutical Sciences | India | Rabies, tuberculosis and trypanosomiasis | Qualitative: interviews |
| 43 | Ozaa & Kulkarnia (2017) | Journal of Pharmacy and Pharmacology | India | Tuberculosis and escherichia coli | Review |
| 44 | Govindarajan et al. (2011) | Asian Pacific Journal of Tropical Medicine | India | Dengue, and lymphatic filariasis | Experimental study |
| 45 | Saxena et al. (2016) | springer | India | Zika virus, dengue and swine fever | Review |
| 46 | Murthy et al. (2010) | Planta Med | India | Lymphatic filariasis | Review |
| 47 | Verma et al. (2013) | BioMed Research International | India | Hydatidosis/cystic echinococcosis | Clinical study |
| 48 | Moudgil et al. (2020) | Veterinarski Arhiv | India | Cystic echinococcosis | In vitro |
| 49 | Palbag et al. (2016) | Indian Journal of Natural Products and Resources | India | Dengue | Review |
| 50 | Zahir et al. (2012) | Experimental Parasitology | India | Leishmaniasis | Experimental study |
| 51 | Amutha et al. (2019) | Pharmacological Benefits of Natural Products | India | Escherichia coli | Review |
| 52 | Rahaman (2011) | African Journal of Pharmacy & Pharmacology | Bangladesh | Escherichia coli | Review |
| 53 | Sonawane et al. (2017) | International Journal of Green Pharmacy | India | Escherichia coli and cholera | In vitro |
| 54 | Manojja et al. (2019) | South African Journal of Botany | India | Brucellosis | In vitro |
| 55 | Das et al. (2015) | Journal of Medicinal Plants Studies | India | Herpes | Review |
| 56 | John et al. (2014) | Saudi Journal of Biological Sciences | India | Avian Influenza | Clinical study |
| 57 | Jayati et al. (2013) | International Journal of Pharmaceutical Sciences | India | Escherichia coli | Clinical study |
| 58 | Prasad et al. (2010) | International Quarterly Journal of Research in Ayurveda | India | Lymphatic filariasis | Experimental study |
| 59 | Pattanaik et al. (2006) | Ethnobotanical Leaflets | India | Cholera | Mixed-methods: survey and interviews |
| 60 | Brijesh et al. (2006) | Journal of Zhejiang University Science B | India | Escherichia coli, salmonella, shigella cholera and campylobacter jejuni | Clinical research |
| 61 | Tyagi et al. (2016) | Journal of Critical Reviews | India | Escherichia coli | Review |
| 62 | Sharma et al. (2019) | International Journal of Green Pharmacy | India | Cholera | Experimental study |
| 63 | Kushwaha et al. (2014) | An International Journal of Research in AYUSH and Allied Systems | India | Cholera | Review |
| 64 | Bora et al. (2016) | Journal of Medicinal Plants Studies | India | Cholera | Household survey |
| 65 | Kumar et al. (2014) | Journal of Parasitology Research | India | Bancroftian Filariasis | Experimental study |
| 66 | Singh et al. (2010) | Journal of Applied Toxicology | India | Schistosomiasis | Review |
| 67 | Kale et al. (2011) | Journal of Chemical and Pharmaceutical Research | India | Lymphatic filariasis, onchocerciasis and schistosomiasis | In vitro |
| 68 | Bhatia et al. (2013) | Indo American Journal of Pharmaceutical Research | India | Escherichia coli | Review |
| 69 | Desai & Desai (2015) | Biotechnological Research | India | Salmonellosis | Experimental study |
| 70 | Padamanabhanathy & Evanjelene (2013) | International Journal of Emerging Technology and Advanced Engineering | India | Salmonella typhi, shigella and escherichia coli | Experimental study |
| 71 | Bhattacharjee et al. (2012) | Asian Pacific Journal of Tropical Biomedicine | India | Rabies | Review |
| 72 | Srivastav & Das (2014) | International Journal of Innovative Research and Development | India | Escherichia coli | Clinical study |
| 73 | Khan (2009) | Advances in Biological Research 3 | India | Salmonella typhi | Clinical study |
| 74 | Singh et al. (2020) | Journal of Scientific Research | Indian sub-continent | Lymphatic filariasis, leishmaniasis, taeniasis and ascariasis | Review |
| 75 | Manohar (2022) | Ancient Science of Life | India | Dengue | Review |
| 76 | Panda et al. (2011) | Journal of Biologically Active Products from Nature | India | Shigella, Salmonella typhi and escherichia coli | Experimental study |
| 77 | Venkateswarlu (2016) | American Journal of Phytomedicine and Clinical Therapeutics | India | Hepatitis | In vitro |
| 78 | Singh & Sharma (2013) | Journal of Research Education in Indian Medicine | India | Hepatitis | Multi-criteria analysis |
| 79 | Anand & Lal (2016) | Journal of Pharmacognosy and Phytochemistry | India | Hepatitis | Review |
| 80 | Appadurai et al. (2015) | Parasite Epidemiology and Control | India | Dengue, chikungunya and Zika virus | Experimental study |
| 81 | Ali et al. (2018) | Parasitology International | India | Dengue and filariasis | Experimental study |
| 82 | Patil &Chaudhary (2016) | International Journal of Green Pharmacy | India | Influenza viruses | Review |
| 83 | Singh et al. (2020) | Journal of Scientific Research | India | Lymphatic fila5riasis, leishmaniasis and taeniasis | Review |
| 84 | Alagesaboopathi (2009) | Complementary and Alternative Medicines | India | Cholera | Mixed methods: survey and interviews |
| 85 | Divyesh et al. (2013) | Drug discovery | India | Escherichia coli | Experimental study |
| 86 | Kalaivani et al. (2012) | Journal of Biologically Active Products from Nature | India | Escherichia coli and shigella | Experimental study |
| 87 | Mariselvam et al. (2014) | Journal of Academia and Industrial Research | India | Shigella | Experimental study |
| 88 | Hajra et al. (2015) | Indian Society for Parasitology | India | Yellow fever and dengue | Clinical study |
| 89 | Yadav et al. (2018) | World Journal of Pharmaceutical Research | India | Dengue | Review |
| 90 | Ghanshyam et al. (2018) | International Journal of Ayush Case Reports | India | Dengue | Case report |
| 91 | Sadhana et al. (2017) | Pakistan Journal of Pharmacology | Pakistan | Escherichia coli and shigella | Review |
| 92 | Chouhan et al. (2015) | Parasites & Vectors | Pakistan | Leishmaniasis | In vivo |
| 93 | Arawwawalaand & Wickramaarachchi (2012) | Pharmacologia3 | Sri Lanka | Influenzas | Laboratory study |
| 94 | Bhattacharya et al. (2013) | Pharmacognosy Research | India | Leishmaniasis | Experimental study |
| 95 | Mekala & KrishnaMurthy (2020) | Journal of Pharmacognosy and Phytochemistry | India | Dengue and chikungunya | Experimental study |
| 96 | Suja et al. (2017) | Journal of Communicable Diseases | India | Tuberculosis | Experimental study |
| 97 | Kaur (2017) | International Journal of Emerging Research in Management &Technology | India | Salmonella typhi and Shigella | Experimental study |
| 98 | Shobi et al. (2018) | Journal of Applied Biotechnology & Bioengineering | India | Escherichia coli, shigella and cholera | Experimental study |
| 99 | Ambrin et al. (2020) | Pakistan Journal of Zoology | Pakistan | Escherichia coli and salmonella typhi | Clinical study |
| 100 | Kaus & Singh (2020) | Asian Journal of Advances in Medical Science | India | Covid-19 | Review |
| 101 | Nath & Yadav (2015) | Indian Society for Parasitology | India | Helminthic infections | In vitro |
| 102 | Ramalingam et al. (2018) | An International Quarterly Journal of Research in Ayurveda | India | Dengue | Review |
| 103 | Uniyal et al. (2014) | Journal of Entomology and Zoology Studies | India | Dengue and chikungunya | Experimental study |
| 104 | Paul et al. (2021) | Journal of Pharmacognosy and Phytochemistry | India | Dengue | Experimental study |
| 105 | Gupta et al. (2020) | International Journal of Tropical Insect Science | India | Dengue, chikungunya and yellow fever | Review |
| 106 | Singh et al. (2020) | Journal of Ayurvedic and Herbal Medicine | India | Covid-19 | Review |

Table S3. Key search terms used across databases

| **Specific search terms** | **Database** | **Number of hits** | **Papers reviewed** | **Date of search** | **Number of relevant papers** | **Number of excluded papers** |
| --- | --- | --- | --- | --- | --- | --- |
| "tick-borne diseases" OR "mite-borne" OR "flea-borne" OR "mosquito-borne diseases" AND "Ayurveda OR "“Traditional healing” OR “Traditional healer” OR healing OR religion OR “Traditional Medicine" AND India (All Fields) and 2021 or 2022 (Exclude – Publication Years) and English (Languages) and INDIA (Countries/Regions) | Web of Science | 10,839 | first 300 | 15/09/2021 | 128 | 172 |
| "Zoonoses" OR "Zoonotic Diseases" AND “Traditional healing” OR “Traditional healing practice” OR “Traditional Medicine” OR “Traditional medicine knowledge" OR "Complementary, and Alternative Medicine" OR "Traditional Indian Medicine" OR "Ayurveda" AND "India" OR "Indian sub-continent" (All Fields) and 2022 or 2021 (Exclude – Publication Years) and English (Languages) and INDIA (Countries/Regions) | Web of Science | 5,733 | first 300 | 15/09/2021 |  |  |
| "Brucellosis" AND "Traditional Medicine" OR "Indian Traditional Medicine" AND "India" OR "Indian Sub-continent" | Web of Science | 752 | first 300 | 15/09/2021 | 14 | 286 |
| "Leptospirosis" AND "Traditional Medicine" OR "Indian Traditional Medicine" AND "India" OR "Indian Sub-continent" | Web of Science | 753 | first 300 | 21/09/2021 | 14 | 286 |
| "tick-borne diseases" OR "mite-borne" OR "flea-borne" OR "mosquito-borne diseases" AND "Ayurveda OR ""Traditional healing" OR "Traditional healer" OR healing OR religion OR " Traditional Medicine" AND India | PubMed | 5,193 | first 300 | 21/09/2021 | 4 | 296 |
| "tick-borne diseases" OR "mite-borne" OR "flea-borne" OR "mosquito-borne diseases" AND "Siddha medicine" OR "alternate medicine" OR "Traditional medicine" AND "India" | Scopus | 1 | 1 | 08/10/2021 | 1 | 0 |
| "Zoonoses" OR "Zoonotic diseases" AND "Traditional healing practice" OR "alternate medicine" OR "Traditional medicine" AND "India" | Scopus | 2 |  | 08/10/2021 |  |  |
| ("Zoonoses" OR "Zoonotic diseases" AND "Traditional healing practice" OR "alternate medicine" OR "Traditional medicine" AND "India" | Scopus | 143 | 143 | 08/10/2021 | 23 | 120 |
| "Leptospirosis" AND "Traditional Medicine" OR "Indian Traditional Medicine" AND "India" OR "Indian Sub-continent" | Scopus | 34 | 34 | 08/10/2021 | 7 |  |
| "Brucellosis" AND "Ayurveda OR "Traditional healing" OR "Traditional healer" OR healing OR religion OR " Traditional Medicine" AND India | PubMed | 12,511 | first 300 | 18/10/2021 | 1 |  |
| "Dengue" AND "Ayurveda OR "Traditional healing" OR "Traditional healer" OR Traditional Medicine" AND India | PubMed | 5,985 | first 300 | 18/10/2021 | 35 | 265 |
| "Zoonoses" OR "Zoonotic diseases" AND "Traditional healing practice" OR "alternate medicine" OR "Traditional medicine" AND "India" | PubMed | 2,415 | first 300 | 01/11/2021 | 23 |  |
